# Increased Plasma Levels of lncRNAs *LINC01268*, *GAS5* and *MALAT1* Correlate with Negative Prognostic Factors in Myelofibrosis

**DOI:** 10.3390/cancers13194744

**Published:** 2021-09-22

**Authors:** Sebastian Fantini, Sebastiano Rontauroli, Stefano Sartini, Margherita Mirabile, Elisa Bianchi, Filippo Badii, Monica Maccaferri, Paola Guglielmelli, Tiziana Ottone, Raffaele Palmieri, Elena Genovese, Chiara Carretta, Sandra Parenti, Selene Mallia, Lara Tavernari, Costanza Salvadori, Francesca Gesullo, Chiara Maccari, Michela Zizza, Alexis Grande, Silvia Salmoiraghi, Barbara Mora, Leonardo Potenza, Vittorio Rosti, Francesco Passamonti, Alessandro Rambaldi, Maria Teresa Voso, Cristina Mecucci, Enrico Tagliafico, Mario Luppi, Alessandro Maria Vannucchi, Rossella Manfredini

**Affiliations:** 1 Centre for Regenerative Medicine, Life Sciences Department, University of Modena and Reggio Emilia, 41125 Modena, Italy; sebastian.fantini@unimore.it (S.F.); sebastiano.rontauroli@unimore.it (S.R.); stefano.sartini@unimore.it (S.S.); margherita.mirabile@unimore.it (M.M.); elisa.bianchi@unimore.it (E.B.); 237834@studenti.unimore.it (F.B.); elena.genovese@unimore.it (E.G.); chiara.carretta@unimore.it (C.C.); sandra.parenti@unimore.it (S.P.); selene.mallia@unimore.it (S.M.); lara.tavernari@unimore.it (L.T.); 2 Department of Laboratory Medicine and Pathology, Diagnostic Hematology and Clinical Genomics, AUSL/AOU Policlinico, 41124 Modena, Italy; maccaferri.monica@aou.mo.it; 3 Department of Experimental and Clinical Medicine, and Center Research and Innovation of Myeloproliferative Neoplasms (CRIMM), University of Florence, Careggi University Hospital, 50134 Florence, Italy; paola.guglielmelli@unifi.it (P.G.); costanzasalvadori@yahoo.it (C.S.); francesca.gesullo@unifi.it (F.G.); chiara.maccari@unifi.it (C.M.); michela.zizza@unifi.it (M.Z.); a.vannucchi@unifi.it (A.M.V.); 4 Department of Biomedicine and Prevention, University of Tor Vergata, 00133 Rome, Italy; tiziana.ottone@uniroma2.it (T.O.); raffaele.f.palmieri@gmail.com (R.P.); voso@med.uniroma2.it (M.T.V.); 5 Santa Lucia Foundation, Istituto di Ricovero e Cura a Carattere Scientifico (I.R.C.C.S.), Neuro-Oncohematology, 00179 Rome, Italy; 6 Department of Biomedical, Metabolic and Neural Sciences, University of Modena and Reggio Emilia, 41125 Modena, Italy; alexis.grande@unimore.it; 7 Hematology, ASST Papa Giovanni XXIII, 24127 Bergamo, Italy; ssalmoiraghi@fondazionefrom.it (S.S.); arambaldi@hpg23.it (A.R.); 8 Division of Hematology, Ospedale ASST Sette Laghi, University of Insubria, 21100 Varese, Italy; barbara.mora@asst-settelaghi.it (B.M.); francesco.passamonti@asst-settelaghi.it (F.P.); 9 Department of Medical and Surgical Sciences, University of Modena and Reggio Emilia, AOU Policlinico, 41124 Modena, Italy; leonardo.potenza@unimore.it (L.P.); enrico.tagliafico@unimore.it (E.T.); mario.luppi@unimore.it (M.L.); 10 Center for the Study of Myelofibrosis, Foundation Policlinico San Matteo, Istituto di Ricovero e Cura a Carattere Scientifico (I.R.C.C.S.), 27100 Pavia, Italy; v.rosti@smatteo.pv.it; 11 Department of Medicine and Surgery, Section of Hematology and Clinical Immunology, University of Perugia, 06129 Perugia, Italy; cristina.mecucci@unipg.it; 12 Center for Genome Research, University of Modena and Reggio Emilia, 41125 Modena, Italy

**Keywords:** lncRNAs, myelofibrosis, MPN, biomarkers, prognosis

## Abstract

**Simple Summary:**

Myelofibrosis (MF) displays the worst prognosis among Philadelphia-negative chronic myeloproliferative neoplasms. There is no curative therapy for MF, except for bone marrow transplantation, which however has a consistent percentage of failure. There is thus an urgent need of novel biomarkers to complement current stratification models and to enable better management of patients. To address this issue, we herein measured the plasma levels of several long noncoding RNAs (lncRNAs). Circulating lncRNAs has been already largely described as potential non-invasive biomarkers in cancers. In our study we unveiled that *LINC01268*, *MALAT1* (both *p* < 0.0001) and *GAS5* (*p* = 0.0003) plasma levels are significantly higher in MF patients if compared with healthy donors, and their increased plasma levels correlate with several detrimental features in MF. Among them, *LINC01268* is an independent variable for both OS (*p* = 0.0297) and LFS (*p* = 0.0479), thus representing a putative new biomarker suitable for integrate contemporary prognostic models.

**Abstract:**

Long non-coding RNAs (lncRNAs) have been recently described as key mediators in the development of hematological malignancies. In the last years, circulating lncRNAs have been proposed as a new class of non-invasive biomarkers for cancer diagnosis and prognosis and to predict treatment response. The present study is aimed to investigate the potential of circulating lncRNAs as non-invasive prognostic biomarkers in myelofibrosis (MF), the most severe among Philadelphia-negative myeloproliferative neoplasms. We detected increased levels of seven circulating lncRNAs in plasma samples of MF patients (*n* = 143), compared to healthy controls (*n* = 65). Among these, high levels of *LINC01268*, *MALAT1* or *GAS5* correlate with detrimental clinical variables, such as high count of leukocytes and CD34+ cells, severe grade of bone marrow fibrosis and presence of splenomegaly. Strikingly, high plasma levels of *LINC01268 (p* = 0.0018)*, GAS5* (*p* = 0.0008) or *MALAT1* (*p* = 0.0348) are also associated with a poor overall-survival while high levels of *LINC01268* correlate with a shorter leukemia-free-survival. Finally, multivariate analysis demonstrated that the plasma level of *LINC01268* is an independent prognostic variable, suggesting that, if confirmed in future in an independent patients’ cohort, it could be used for further studies to design an updated classification model for MF patients.

## 1. Introduction

The Philadelphia (Ph)-negative myeloproliferative neoplasms (MPNs) are clonal stem-cell disorders characterized by an increased proliferation and abnormal differentiation of myeloid cells. They include polycythemia vera (PV), essential thrombocythemia (ET) and primary myelofibrosis (PMF), the latter identified as prefibrotic (pre-PMF) and overtly fibrotic (overt-PMF) stages according to the degree of bone marrow fibrosis [1]. In addition to PMF, which has a de novo onset, secondary myelofibrosis (SMF) might evolve from PV or ET, resulting in post-PV myelofibrosis (PPV-MF) or post-ET myelofibrosis (PET-PV), respectively, all generally referred to as myelofibrosis (MF) [2].

MPNs originate from somatic mutations occurring in hematopoietic stem cells. Mutations in *JAK2*, *MPL* and *CALR* genes have been identified as driver events of disease onset, although observed with different frequencies in the specific diseases [3]. In addition, “High Molecular Risk (HMR)” mutations (e.g., in *ASXL1*, *IDH1/2*, *SRSF2* and *EZH2* genes) have been associated to a worse prognosis and a more frequent leukemic transformation [4]. The serial acquisition of somatic mutations underlies the clonal evolution in MPNs and we have recently reconstructed by single cell analysis the sequence of mutational events associated to PMF progression [5]. MF is the most severe among the MPNs and is characterized by the occurrence of bone marrow fibrosis and a consequent abnormal increase in the number of circulating CD34+ hematopoietic progenitors [6]. The most frequent clinical manifestations of MF include splenomegaly, caused by extramedullary hematopoiesis, bleedings and constitutional symptoms. Besides infections, thrombosis and cardiovascular complications, the main cause of death is due to transformation to acute myeloid leukemia (AML), which occurs in 15–20% of cases and is not responsive to conventional therapeutic treatments [4].

Prediction of patients’ survival, with the aim to tailor the best therapeutic approach, is based on current prognostic models, such as the International Prognostic Scoring System (IPSS) [7], dynamic-IPSS (DIPSS) [8] and the more recently developed Mutation-enhanced International Prognostic Score System (MIPSS70) [9] and MIPSS70+ version 2.0 [10,11]. Although allogenic stem cell transplantation (ASCT) is currently the only curative therapeutic approach for MF, it is still associated to frequent graft-related morbidities and deaths [12]. Thus, it is of great interest to establish new specific prognostic factors that may predict disease outcome to identify high risk patients eligible for ASCT. In this regard, we have recently proposed that gene expression profiles in MF granulocytes might integrate current prognostic models [13].

Long non-coding RNAs (lncRNAs) are RNA molecules longer than 200 nucleotides and lacking protein-coding potential. It has been demonstrated that lncRNAs are heavily involved in several cellular mechanisms, inter alia, the regulation of gene expression, either in cis or in trans, chromatin remodeling, RNA processing and organization of nuclear domains. They have been found embedded as key components in highly wired networks of gene expression regulation [14]. LncRNAs expression is finely regulated, displaying even higher tissue and cell specificity than protein-coding mRNAs and making them candidate biomarkers and potential targets for therapeutic approaches [15,16].

An exhaustive description of the roles of lncRNAs in normal and malignant hematopoiesis has yet to be provided. However, it has been demonstrated that they play a pivotal role in the differentiation processes within specific hematopoietic lineages (e.g., *EGO* in eosinophils commitment [17], *HOTAIRM1* in RA-induced granulocytic differentiation [18]). In many cases, a dysregulation in a single lncRNAs expression has also been linked to blood malignancies development and progression [19,20].

Over the past decade, evidence has been accumulating about the detection of RNAs in biofluids and their putative application as biomarkers in the early diagnosis and prediction of prognosis and therapy response for cancer patients [21]. Circulating RNAs are secreted from cells through active processes or released after cell death [22]. Notably, lncRNAs might be extensively detected in body fluids, despite of their low levels of expression, since they form secondary structures that confer them high stability [23].

In the present study we took to explore the expression profile of circulating lncRNAs in plasma samples from MF patients, with the aim to assess their potential significance as prognostic indicators of disease progression in the light of performing personalized therapeutic approaches. We identified by quantitative reverse transcription PCR (qRT-PCR) increased levels of circulating *LINC01268*, *MALAT1* and *GAS5* in MF patients and we correlated their expression with the patients’ clinical features and prognosis.

## 2. Materials and Methods

### 2.1. Patients

Human CD34+ cells were purified by using magnetic beads separation system (CD34 MicroBead Kit UltraPure, Miltenyi Biotech, Auburn, CA, USA) from peripheral blood (PB) of 83 patients (MFs). As control samples, CD34+ cells were isolated from 14 PB samples and 12 bone marrow (BM) samples from healthy donors (HDs).

Plasma samples were separated from 65 HDs and 143 patients with a diagnosis of PMF or SMF, recruited from seven Italian research centers. PMF was diagnosed according to 2016 World Health Organization criteria [1], whereas International Working Group for Myeloproliferative neoplasms Research and Treatment criteria were exploited for the diagnosis of PET-MF and PPV-MF [2]. This study was conducted in accordance with the Declaration of Helsinki and after the approval by local ethics committees. All subjects provided informed written consent.

### 2.2. Plasma Isolation

Blood samples were collected via venipuncture in EDTA or sodium citrate containing tubes. Plasma was separated from blood cells by centrifugation at 1900× *g* at room temperature for 5 min. Cell debris were removed with a second centrifugation at 17,000× *g* at +4°C for 5 min. Hemolyzed samples were identified by spectrophotometric analyses, measuring the absorbance of hemoglobin at 414 nm using the NanoDrop ND-1000 spectrophotometer (ThermoFisher Scientific, Waltham, MA, USA). Samples with a hemoglobin absorbance ≥ 0.3 were considered hemolyzed and excluded from further analyses [24]. Plasma was then aliquoted and stored at −80 °C.

### 2.3. RNA Purification

Total RNA from CD34+ cells was extracted using the miRNeasy Micro Kit (Qiagen, Hilden, Germany) following manufacturer’s instructions.

For plasma analysis, total RNA purification was obtained from 100 μL of plasma samples by using the MagMAX^TM^ mirVana^TM^ Total RNA Isolation Kit (Applied Biosystems, ThermoFisher Scientific, Waltham, MA, USA) according to the manufacturer’s specifications for plasma samples. Samples were thawed at room temperature to avoid the formation of cryoprecipitates. Synthetic spike-in RNA (#A39179, Taqman Universal RNA spike In reverse Transcription control, Applied Biosystems, Waltham, MA, USA) was introduced in each sample during RNA extraction as an exogenous control. Total RNA was eluted in a final volume of 30 μL and then stored at −80 °C.

The recovery of total RNA obtained with this protocol was too low to evaluate RNA quantity and integrity by spectrophometric or fluorimetric measurements. For this reason, the following steps were performed starting from the same volume of RNA elution for each sample.

### 2.4. Measuring lncRNAs Expression in CD34+ Cells

Reverse transcription reaction was performed starting from 100 ng of total RNA from CD34+ cells by using Superscript^TM^ VILO^TM^ cDNA Synthesis Kit (Applied Biosystems, ThermoFisher Scientific, Waltham, MA, USA) according to the manufacturer’s instructions.

Next, a preamplification step was run mixing 5 μL of cDNA with pooled Taqman assays and TaqMan^TM^ PreAmp Master mix (Applied Biosystems, ThermoFisher Scientific, Waltham, MA, USA) to increase uniformly the amount of cDNA for each target lncRNAs. Pooled Taqman assays mix was prepared by combining equal volumes of assays for all the targets of interest (listed in Table S1) so that each assay is at a final concentration of 0.2×.

Amplification reactions were performed on Applied Biosystems^TM^ QuantStudio^TM^ 12K Flex Real-Time PCR System (ThermoFisher Scientific, Waltham, MA, USA). We exploited custom OpenArray plates (56-assays format) both with custom and predesigned Taqman assays. Each OpenArray plate allowed simultaneous detection of lncRNAs of 16 samples in triplicate. For each sample, pre-amplified cDNA was diluted 1:20, mixed with TaqMan^TM^ OpenArray™ Real-Time PCR Master Mix and loaded onto OpenArray™ plate by means of QuantStudio™ OpenArray^®^ AccuFill™ System.

Data analysis was performed by means of Relative Quantification App on ThermoFisher Cloud (ThermoFisher Scientific, Waltham, MA, USA) applying the C_RT_ method (“relative threshold” method). The C_RT_ method, made necessary by the small volume loaded on each through-holes, differs from the common C_T_ method since it defines a threshold for each reaction based on the efficiency of reaction. We included, for further analyses, all the reactions with a C_T_ ≤ 30 and good amplification scores (Amp Score > 1.0, Cq confidence > 0.8). Expression data have been analyzed applying the ΔΔC_T_ method [25], using the geometric mean of four endogenous controls (*RPL19*, *IPO8*, *HPRT1* mRNAs and *LINC01353* non-coding RNA) as normalization factor and the median ΔCt score of the value of HDs as calibrator. Stability expression of candidate reference controls among all the analyzed samples was evaluated by means of GeNorm algorithm [26]. Fold change (FC) expression for each lncRNA was calculated by using the 2^−ΔΔCT^ method comparing MF plasma samples with HDs.

### 2.5. Measuring lncRNAs Levels in Plasma

Sample workflow to detect lncRNAs in plasma followed the same steps above described, with the following modifications.

Reverse transcription reaction was performed starting from 10 μL of total RNA prepared from plasma samples. Amplification reactions were performed in duplicate for each sample, using the TaqMan^TM^ Fast Advanced Master Mix and predesigned TaqMan probes specific for each target (listed in Table S2) on the AB 7900HT Fast Real-Time PCR System (Applied Biosystems, Waltham, MA, USA).

The evaluation of the previously added synthetic spike-in RNA was run simultaneously to assess the efficiency of purification and reverse transcription processes.

For data analysis we included all the reactions with a C_T_ < 33, and maximum cycle value (C_T_ = 40) was attributed to amplification reactions with C_T_ ≥ 33. Expression data have been analyzed applying the ΔΔC_T_ method [25], using *H19* as endogenous control since resulted as the lncRNA more stably expressed between control and MF groups. In addition, it displayed a distribution in the two groups similar to miR-23a, a small RNA previously reported as reference for circulating RNAs (Figure S1) [24]. On the contrary, *GAPDH*, *HPRT1* and *ACTB* resulted significantly more enriched in MF plasma samples and, also being coding mRNAs, are thus excluded as endogenous controls.

The median ΔCt value of HDs was exploited as calibrator. FC expression for each lncRNA was calculated by using the 2^−ΔΔCT^ method comparing MF plasma samples with HDs.

### 2.6. Statistical Analysis

Comparisons between two groups of numerical variables were performed by using the non-parametric Mann–Whitney U Test. Fisher’s exact test or the Chi-square (χ^2^) test was performed to discriminate differences between categorical variables. Outliers were removed from further analyses by the ROUT method. Overall survival (OS) and leukemia-free survival (LFS) were calculated from the date of sample collection to the date of last follow-up or event occurrence (death or leukemic transformation, respectively). OS and LFS analyses were performed with the Kaplan–Meier method, and the log-rank test was used to compare two curves. Multivariate analyses were carried out by means of Cox proportional hazard regression for OS and LFS using R version 3.4.1. *p*-values (*p*) < 0.05 were considered as statistically significant. All graphs and statistical analyses were performed using GraphPad Prism version 8.4.0 for Mac (GraphPad Software, San Diego, CA, USA). PCA and hierarchical clustering were performed using Partek Genomics Suite (GS) software, version 7.0 (Partek Inc., St. Louis, MO, USA).

## 3. Results

### 3.1. LncRNAs Expression Profile in CD34+ Cells from MF Patients

**Figure 1:**
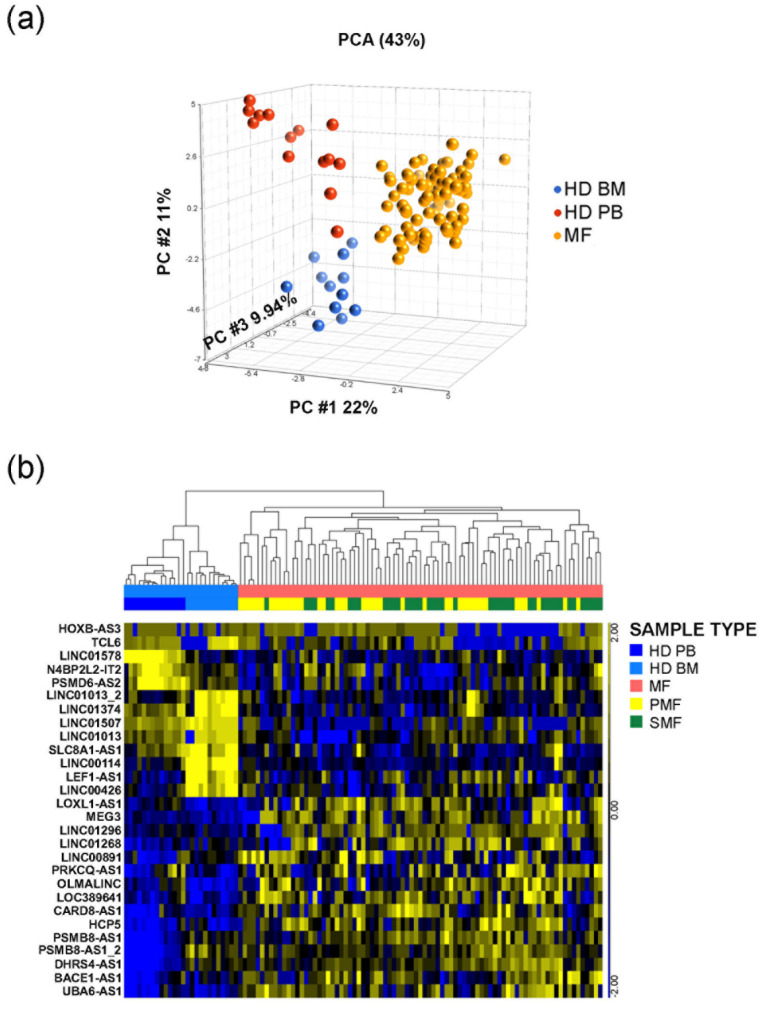
PCA and Hierarchical clustering of OpenArray Data. (**a**) PCA graph represents the global lncRNAs expression in CD34+ stem/progenitor cells. (**b**) Hierarchical clustering of samples according to the expression of 28 lncRNA transcripts, corresponding to 26 lncRNAs. Both analyses were performed by Partek GS, version 6.6. FDR = False Discovery Rate; BM = control samples isolated from bone marrow; PB = control samples isolated from peripheral blood; HD = healthy donor; MF = myelofibrosis patients; PMF = primary myelofibrosis; SMF = secondary myelofibrosis.

Eighty-three MF patients (n = 41 PMF, n = 26 PET-MF, n = 16 PPV-MF) and twenty-six HDs were analyzed for the expression of a list of 38 lncRNAs (Figure 1 and Table S1) by OpenArray real-time PCR platform. Some of the targets have been addressed by using multiple assays to obtain the higher coverage of all their different isoforms. In order to display relationship between samples, we performed unsupervised Principal Component Analysis (PCA) of expression data that demonstrated that MF samples clustered together and clearly differed from healthy controls (Figure 1a). By ANOVA analysis, we identified 26 differentially expressed lncRNAs with a FC > |1.5| in MF samples compared to HD controls. According to hierarchical clustering analysis (FDR < 0.05), these differentially expressed lncRNAs clearly separated our data set into two main branches, one of them including control samples and the other containing all MF patients, respectively. Samples from PMF and SMF patients resulted interspersed and did not clearly separate (Figure 1b).

Together, these results depicted the expression profile of lncRNAs in CD34+ cells and showed that some lncRNAs (such as *MEG3*, *LINC01268*, *TCL6*, *LINC01296*) are differentially expressed in samples isolated from MF if compared with their normal counterparts.

### 3.2. Seven Circulating lncRNAs Are Increased in MF Patients

According to OpenArray results, *LINC01268*, *HOXB-AS3*, *MEG3* and *TCL6* transcripts were selected since deregulated in CD34+ stem/progenitor cells; further lncRNAs have been selected for their associations with myeloid differentiation and deregulation in blood neoplasms [20,27,28,29,30,31,32,33,34]. Plasma levels of thirteen lncRNAs were evaluated in 65 HDs and 143 MF patients (n = 97 PMF, n = 25 PET-MF, n = 21 PPV-MF) using real time qRT-PCR. A complete list of the lncRNAs and of the isoforms covered by Taqman assays used in qRT-PCR experiments is shown in Table S2. All targets detected in fewer than half of plasma samples of MF patients (*TCL6*, *HOXB-AS3*, *MEG3* and *MIR-155HG*) were excluded from further analyses since considered not indicative (data not shown).

**Figure 2:**
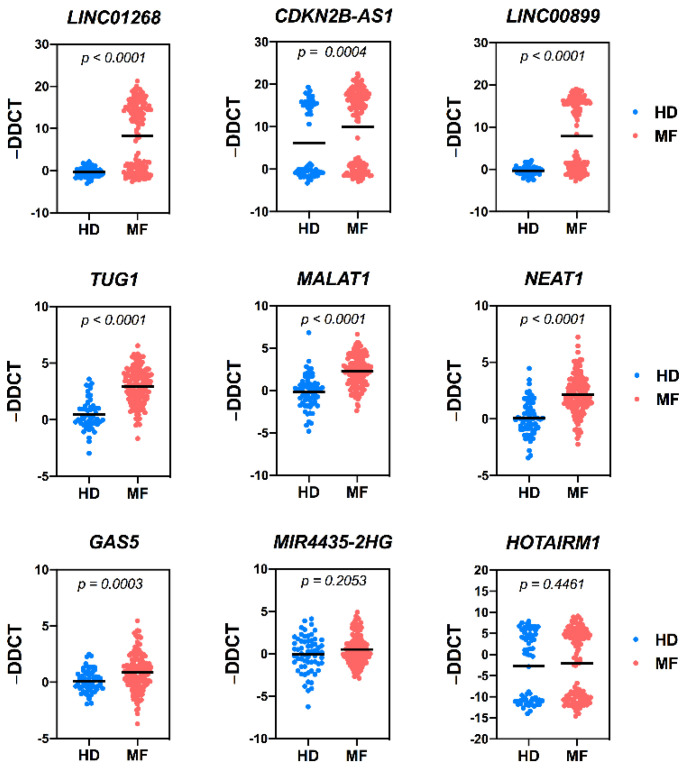
Circulating levels of lncRNAs in HD and MF samples. Scatter dot plots represent the circulating plasma levels of selected lncRNAs in control (HD) and MF samples. Data are expressed as −ΔΔCT using *H19* lncRNA as endogenous control and the median ΔCT of the group of control samples as calibrator. Control samples are represented by blue dots, whereas MF sample by red dots. The black horizontal line in middle represents the median value. *p* = *p*-value computed by non-parametric Mann–Whitney U test. N = 65 for control samples; n = 143 for MF samples. HD = healthy donor, MF = myelofibrosis samples.

The plasma levels of the remaining nine circulating lncRNAs are shown in Figure 2, whereas the relative FC is reported in Table S2. Several circulating RNAs were assessed as putative reference endogenous controls. Among them, *H19* was selected since recovered at similar levels in control and MF plasma samples. Moreover, its distribution mirrors the pattern displayed by RNAs (i.e., hsa-miR-23a) previously reported as endogenous control for circulating RNAs [24] (Figure S1). A strong increase of circulating *LINC01268* (FC = 2.164, *p* < 0.0001) and *CDKN2B-AS1* (FC = 22,011 and *p* = 0.0004) was detected in MF samples if compared to HDs, confirming the expression data obtained in CD34+ cells and the results of Pennucci et al. [20]. Furthermore, a significant increase of *LINC00899* (FC = 9.57, *p* < 0.0001), *TUG1* (FC = 7.89, *p* < 0.0001), *MALAT1* (FC = 5.23, *p* < 0.0001), *NEAT1* (FC = 4.04, *p* < 0.0001) and *GAS5* (FC = 1.82, *p* < 0.0003) was also detected. No statistical difference was found in *MIR-4435-2HG* and *HOTAIRM1* expression between HD and MF samples (Figure 2).

Therefore, seven lncRNAs were selected as potential biomarkers in MF patients and tested for correlations with patients’ clinical and molecular features.

### 3.3. Increased Plasma Levels of LINC01268, MALAT1, GAS5, LINC00899 and TUG1 lncRNAs Correlate with Clinical Detrimental Features

We investigated the potential association between features considered detrimental for MF progression and the seven lncRNAs whose plasma levels has been found increased in MF samples.

In order to unveil potential association between a number of detrimental clinical features and high plasma levels of circulating lncRNAs, the patient cohort was split into two groups (low- or high-) according to the lncRNAs levels. For *LINC01268*, *LINC00899* and *CDKN2B-AS1*, displaying a bimodal distribution within MF samples (Figure 2 and Figure S2), a cutoff value was arbitrarily set between the two peaks. For the remaining target RNAs (*MALAT1*, *GAS5*, *TUG1*, *NEAT1*), displaying a normal distribution, the median value among the MF samples was used as cutoff according to the “median split” method (Figure 2 and Figure S2). A good correlation with detrimental clinical variables was observed for *LINC01268*, *MALAT1*, *GAS5*, *LINC00899* and *TUG1* (Table 1), whereas *NEAT1* and *CDKN2B-AS1* levels seems to be less related to MF prognosis (Table S3).

**Figure 3:**
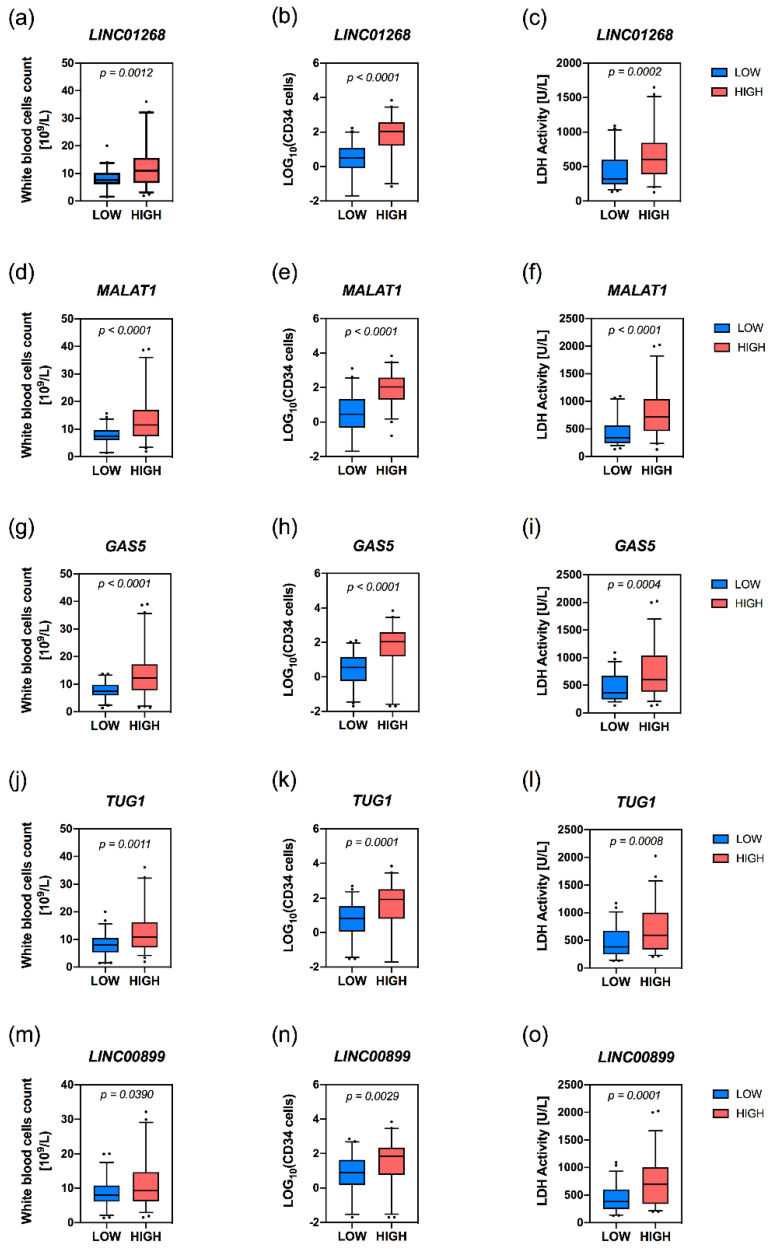
Correlation analysis of level of circulating lncRNAs with WBC count, circulating CD34+ count and LDH activity in MF patients. Box plots represent values from white blood cell count (WBC) (**a**,**d**,**g**,**j**,**m**), CD34+ cells count (**b**,**e**,**h**,**k**,**n**) and LDH activity (**c**,**f**,**i**,**l**,**o**) in MF samples presenting low or high levels of target RNA. The box extends from the 25th to the 75th percentiles. The line in middle of the box is plotted as the median. The whiskers are drawn as 5th and 95th percentiles. Values above or below the whiskers are plotted as individual points. Samples with low or high levels of target lncRNA are represented by blue and red boxes, respectively. *p* = *p*-value was computed by non-parametric Mann–Whitney U test.

Higher plasma levels of LINC01268, MALAT1, GAS5, TUG1 and LINC00899 lncRNAs were associated with elevated white blood cells (WBCs), circulating CD34+ cells together with a marked lactate dehydrogenase (LDH) plasmatic activity (Figure 3a–o).

An increased plasma level of circulating MALAT1 or GAS5 was associated with a lower median hemoglobin level (*p* = 0.0139 and *p* = 0.0072, respectively) and a decrease in the median hematocrit value is displayed only by the group with high levels of MALAT1. Platelets median count was lower in patients with high levels of circulating LINC01268 (*p* = 0.0021) and GAS5 (*p* < 0.0001). The presence of constitutional symptoms was significantly associated with higher plasma levels of MALAT1 (*p* = 0.0302) or GAS5 (*p* = 0.0306), whereas splenomegaly was correlated to higher LINC01268 (*p* = 0.0012), MALAT1 (*p* < 0.0001) or GAS5 levels (*p* = 0.0006) (Table 1).

**Figure 4:**
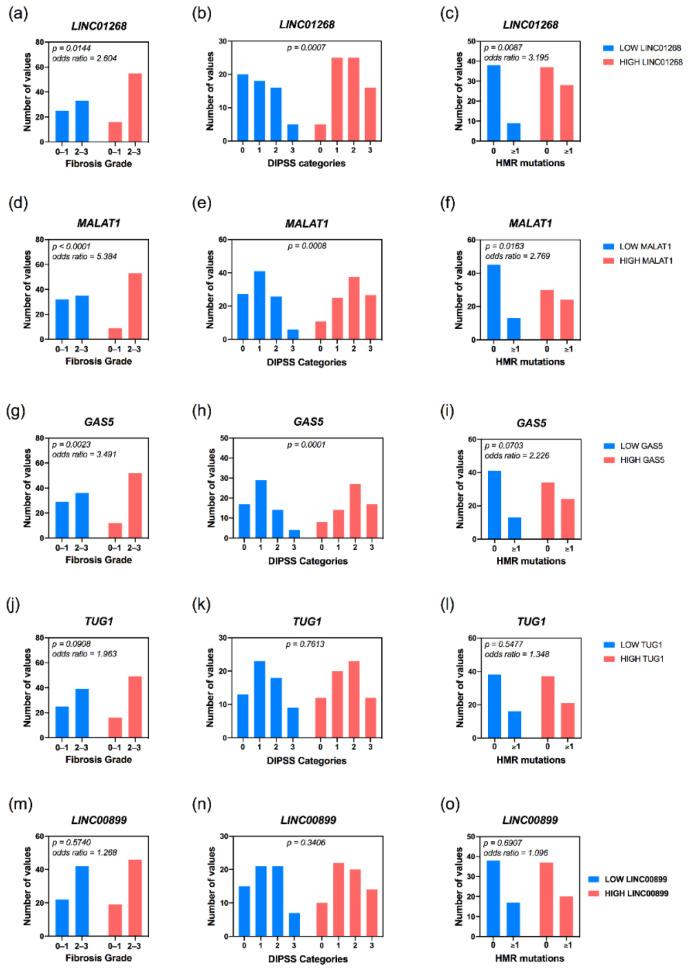
Correlation analysis of level of circulating lncRNAs with fibrosis and mutational status of MF patients. Histograms obtained from contingency tables computed to correlate lncRNAs plasma levels and prognostic estimates such as fibrosis grade (**a**,**d**,**g**,**j**,**m**), assignment to DIPSS categories (**b**,**e**,**h**,**k**,**n**) and HMR mutations (**c**,**f**,**i**,**l**,**o**). Samples with low or high levels of target RNA are represented in blue and red, respectively. *p* = *p*-value was computed by Fisher’s exact test or the Chi-square (χ^2^) test.

MALAT1 and GAS5 have been found associated to MF clinical subtypes (*p* = 0.0001 and *p* = 0.0204, respectively), with pre-PMF samples enriched within the group of patients with low levels of circulating MALAT1; on the contrary, high levels of GAS5 correlated with patient with overt-PMF (Table 1). Accordingly, MALAT1 (*p* = 0.0004) and GAS5 (*p* = 0.0023) levels positively correlate with a more severe grade of bone marrow fibrosis. Furthermore, high levels of LINC01268 (*p* = 0.0144) are also associated to a more marked grade of fibrosis (Figure 4a,d,g).

Notably, an interesting correlation emerged between circulating lncRNAs and DIPSS prognostic score system. Patients belonging to DIPSS Intermediate-2 and High categories being enriched in high LINC01268 (*p* = 0.0007), MALAT1 (*p* = 0.0008) and GAS5 (*p* = 0.0001) groups, whereas the majority of patients classified as DIPSS Low are gathered in groups with low plasma levels of lncRNAs (Figure 4b,e,h).

Taken together, these data highlighted the association between clinical features commonly monitored in MF patients and circulating levels of several lncRNAs. In particular, we unveiled that LINC01268, MALAT1 and GAS5 are the lncRNAs more frequently associated to clinical detrimental features in MF patients and thus of great interest as putative markers of MF prognosis.

### 3.4. High Plasma Levels of LINC01268, MALAT1, GAS5, TUG1 and NEAT1 Are Associated with MF Patients’ Molecular Features

We selected a cohort of patients displaying expected frequencies in driver mutations distribution: JAKV617F was the most common mutation, occurring in 61% of samples, MPL in 6%, CALR in 27%, whereas 6% of patients are triple negative. Mutation in the CALR gene was the sole driver mutation differentially distributed, being enriched in patients with high plasma levels of LINC01268 (*p* = 0.0184), TUG1 (*p* = 0.0311) and NEAT1 (*p* = 0.0059). 

On the other hand, regarding HMR mutations, the frequency of gene variants in ≥1 HMR gene was increased in patients with high levels of LINC01268 (*p* = 0.0087) and MALAT1 (*p* = 0.0163) (Figure 4c,f). Furthermore, occurrence of ≥2 HMR mutations seemed to be associated with LINC01268 levels, being detected only in patients displaying high levels of circulating LINC01268. 

In particular, ASXL1 gene variants were more frequent in patients with high levels of LINC01268 (*p* = 0.0025), MALAT1 (*p* = 0.0101) as well as GAS5 (*p* = 0.0183), while SRSF2 variants were correlated with low levels of NEAT1.

### 3.5. High Plasma Levels of LINC01268, GAS5 and MALAT1 Affect OS

**Figure 5:**
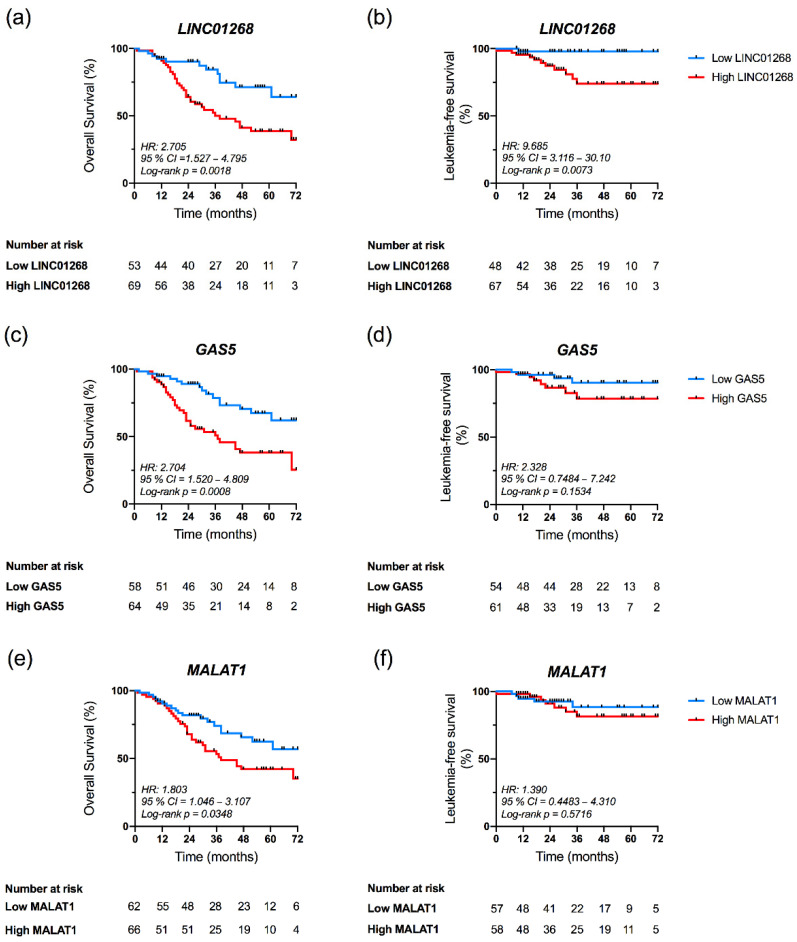
Kaplan–Meier analysis of Overall Survival (OS) and Leukemia-free Survival (LFS) according to LINC01268, GAS5 and MALAT1 plasma levels. Kaplan–Meier estimates of OS (**a**,**c**,**e**) and LFS (**b**,**d**,**f**) of MF patients in the study. Patients’ cohort was stratified into two groups (low and high) according to the plasma levels of target lncRNA, as described in the text. Differences between two survival curves was evaluated by Log-rank (Mantel–Cox) test. Blue and red curves represent patients with low or high levels of circulating target, respectively. HR = hazard ratio computed to determine the magnitude of differences between two curves; *p*-value was computed by log-rank test; 95% CI = 95% confidence interval.

Since patients with high levels of some circulating lncRNAs displayed several detrimental features, we evaluated the association between plasma levels of these seven lncRNAs and OS. The Kaplan–Meier estimates demonstrated that patients with high LINC01268 had an inferior survival rate compared with patients with low LINC01268 (*p* = 0.0018, log-rank test; hazard ratio (HR) = 2.705) (Figure 5a). In particular, OS at six years of follow up was 32.1% and 64% in the group of high or low LINC01268, respectively (data not shown). Similarly, patients with high GAS5 plasma levels displayed an inferior survival compared to patients with low levels (*p* = 0.0008, log-rank test; HR = 2.704) (Figure 5c), with survival proportion of 25.4% and 62%, respectively (data not shown). A statistically significant difference in OS was also observed between groups dichotomized according to circulating levels of MALAT1 (*p* = 0.0348, log-rank test; HR = 1.803) (Figure 5e). No differences were instead observed in OS of MF patients according to plasmatic levels of TUG1, LINC00899, NEAT1 and CDKN2B-AS1 (Figure S3a,c,e,g). In agreement, correlation analysis indeed revealed that, within groups displaying high levels of LINC01268 (*p* = 0.0035), GAS5 (*p* = 0.0115) and MALAT1 (*p* = 0.0436), deaths resulted more frequent. (Table 1 and Table S3).

A similar approach was exploited to assess association between circulating lncRNA levels and LFS. Kaplan–Meier curves showed that high levels of LINC01268 associated with a shorter LFS compared to patients with lower levels (*p* = 0.0063, log-rank test; HR = 9.685) (Figure 5b). No correlation with LFS was instead observed for MALAT1 and GAS5 levels (*p* = 0.5716 and *p* = 0.1534, respectively, log-rank test) (Figure 5d,f) or for the remaining lncRNAs studied (Figure S3b,d,f,h).

Together these results demonstrated that circulating lncRNAs LINC01268, GAS5 and MALAT1, all detected at higher levels in MF patients if compared to HDs and associated to several features detrimental for MF prognosis, correlated with a shorter OS.

### 3.6. LINC01268 Plasma Level Is an Independent Prognostic Factor for OS and LFS

As mentioned before, we observed that plasma levels of LINC01268, MALAT1 and GAS5 correlated with DIPSS classification of MF patients (Figure 4). In order to evaluate the independent prognostic value of these lncRNAs we performed a DIPSS-adjusted multivariate analysis.

**Figure 6:**
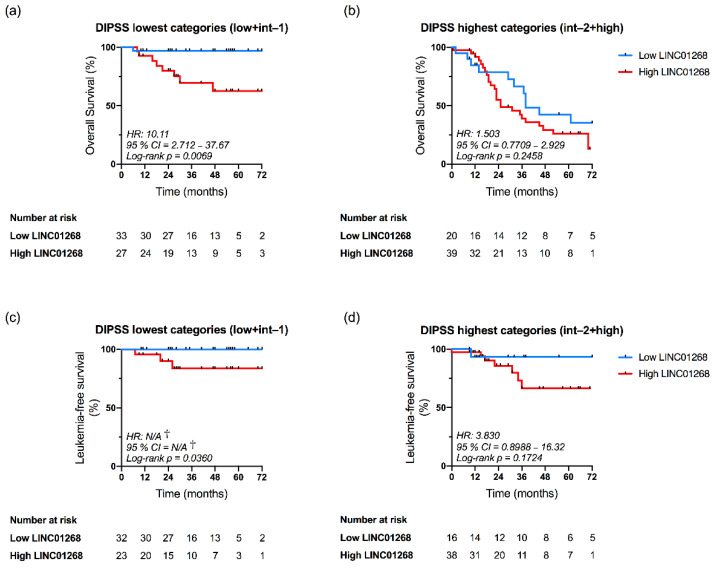
Kaplan–Meier analysis of Overall Survival (**a**,**b**) and Leukemia-free Survival (**c**,**d**) in the settings of DIPSS categories according to LINC01268 plasma levels. Patients were dichotomized in two subgroups according to DIPSS classification: lowest categories (**a**,**c**) correspond to Low and Intermediate-1 classes while highest categories (**b**,**d**) include Intermediate-2 and High groups. Patients’ cohort was then stratified according to low and high plasma levels of LINC01268, as previously described in the text. Differences between survival curves were evaluated by Log-rank (Mantel–Cox) test. Blue and red curves represent patients with low or high levels of circulating LINC01268, respectively. HR = hazard ratio computed to determine the magnitude of differences between two curves. † = No event occurred in the Low LINC01268 subgroup; *p*-value was computed by log-rank test; 95% CI = 95% confidence interval.

Kaplan–Meier curves demonstrated that OS was significantly reduced in patients displaying high plasma level of LINC01268 when considering DIPSS lowest-risk categories (Low and Intermediate-1) (*p* = 0.0069, log-rank test; HR = 10.11) (Figure 6a), but not DIPSS highest categories (Intermediate-2 and High) (Figure 6b). Moreover, multivariate analysis confirmed the independent prognostic relevance for OS of LINC01268 plasma level when considering DIPSS classification (HR = 2.104; confidence interval (CI) = 1.08–4.12; *p* = 0.0297) (Table 2). Similarly, LFS was reduced in the high LINC01268 group only in lowest DIPSS categories (*p* = 0.0360, log-rank test) (Figure 6c,d) and multivariate analysis confirmed the negative impact of higher LINC01268 on LFS independent from DIPSS prognostication (HR = 8.190; CI = 1.02–65.78; *p* = 0.0479) (Table 2). Conversely, our analysis was not able to highlight an independent prognostic relevance for MALAT1 and GAS5 (Table 2).

Taken together, results of multivariate analysis demonstrated that LINC01268 plasma level in MF patients is an independent factor for both OS and LFS analysis.

## 4. Discussion

Myelofibrosis is a hematological neoplasm which originates from somatic mutations emerging in the hematopoietic stem cells compartment. Once occurring in the *JAK2*, *MPL* and *CALR* genes, these mutations might be considered as driver events. However, about 5% of patients display none of them and are thus classified as triple negative. A number of other mutations might be found in MF patients, such as those affecting *ASXL1*, *IDH1/2*, *EZH2* and *SRSF2* genes referred to as HMR, since these are associated with a diminished OS and/or LFS [35]. According to its onset, MF might be primary or secondary when progressed from PV and ET. In addition, a pre-fibrotic and an overt stage might be identified within PMF according to the degree of bone marrow fibrosis [1]. Although establishing different entities, they are managed following the same therapeutic strategies and ASCT is considered so far as the sole curative treatment, even if it is still associated to frequent graft-related deaths and comorbidities [12]. Thus, the identification of specific prognostic predictors, such as new biomarkers, might integrate current prognostic scoring systems [8,9,10] to enable a better management of MF patients.

In this light, expression profiling studies have been extensively performed in recent years in order to provide new signatures suitable to integrate or substitute standard prognostic and/or predictive factors for cancer patients. In particular, gene expression pattern has been exploited to discover new additional prognostic indicators and to identify the best therapy for breast cancer [36], ovarian cancer [37] and melanoma [38]. A 17-gene signature was also proposed to predict survival in AML [39]. Recently, we demonstrated that gene expression profiling of granulocytes provides complementary prognostic information to manage MF patients [13].

MF is characterized by an intricated interplay between malignant hematopoietic stem cells and stromal cells in the bone marrow microenvironment, as synthesized by the “bad seeds in bad soil” concept. Several types of molecules have been identified as involved in this crosstalk, such as cytokines and growth factors together with oxygen and calcium deregulation [40]. By contrast, an exhaustive characterization of the putative involvement of circulating nucleic acids (CNAs) in MF pathogenesis has not been provided so far.

The fibrotic conversion of bone marrow occurring in MF induces a release into circulation of CD34+ cells normally residing in bone marrow [41]. Circulating neoplastic cells derived from neoplastic clones might thus discharge an abnormal number of secreted molecules into plasma.

In order to assess the potential significance as prognostic indicators of disease progression, in this study we decided to measure the levels of circulating lncRNAs since their secondary structures confer a high stability in body fluids [23]. In fact, due to their high specific pattern of expression and functional diversity in a variety of solid and hematological disorders, lncRNAs have promising application in cancer diagnosis, prognosis and therapy.

The presence of RNA molecules in plasma was firstly described in 1999 [42,43], but several factors, such as their low abundance in liquid samples, have prevented their adoption as cancer biomarkers for a long time. Circulating small non-coding RNAs (i.e., microRNAs) are more frequently investigated in research works since interaction with AGO2 protein preserves them from RNase activity [21].

Notably, despite the increasing number of publications about the putative utility of circulating non-coding (ncRNAs) as clinical biomarkers, urinary lncRNA *PCA3* is the only circulating RNA approved by the FDA for molecular testing and used to complement PSA for management of early prostate cancer [44].

This is the first report in which plasma levels of several lncRNAs have been assessed in MF patients. To this purpose, we selected a list of 13 lncRNAs deregulated in hematological malignancies both from literature and from preliminary gene-expression data in CD34+ cells. Among them, we identified seven lncRNAs, namely *LINC01268*, *GAS5*, *MALAT1*, *LINC00899*, *TUG1*, *NEAT1*, *CDKN2B-AS1*, as increased in plasma of MF patients if compared with healthy controls. Our results clearly demonstrated that patients’ stratification based on plasma levels of these circulating lncRNAs are in agreement with the presence of detrimental prognostic factors. In particular, high levels of *LINC01268*, *GAS5* and *MALAT1*, correlated with detrimental features of MF, such as high WBC count, increased number of circulating CD34+ cells, marked LDH activity, presence of splenomegaly and a more severe grade of bone marrow fibrosis. By contrast, low levels of these lncRNAs correlate with the absence of these detrimental features and with a better OS, as described in [45]. Moreover, the percentage of patients displaying ≥1 HMR mutation was increased in group of patients displaying high levels of *LINC01268* or *MALAT1* and, more specifically, patients harboring *ASXL1* mutation are enriched in the group with high levels of *LINC01268*, *MALAT1* or *GAS5*. 

Strikingly, the group of patients with high *GAS5*, *MALAT1* or *LINC01268* were characterized by a shorter OS, the latter leading in addition to a worse LFS. Once stratified according to DIPSS scoring system, patients classified into the High category were more frequently characterized by high levels of *LINC01268*, *GAS5* or *MALAT1* while low levels of these lncRNAs were more frequent in DIPSS Low class. Therefore, we demonstrated that lncRNA plasma levels correlated with contemporary prognostic scoring system. In order to evaluate whether lncRNAs might provide additional independent prognostic information we performed multivariate analysis. Our results demonstrated that high plasma levels of *LINC01268* was also able to predict a shorter OS and LFS independently from the DIPSS classification, in particular when considering DIPSS lowest-risk categories (Low and Intermediate-1). This might be important because only observation or palliative therapy in the presence of specific symptoms is suggested for patients belonging to DIPSS Low or Intermediate-1 classes. More effective treatments, such as ASCT, are to be considered only for patients belonging to Intermediate-2 or High risk categories [46]. Our results suggest that evaluation of *LINC01268* plasma levels might be used to identify patients at higher risk within DIPSS lowest categories thus guiding more appropriate clinical decision.

From this study, it emerges that *GAS5*, *MALAT1* and *LINC01268* are differentially expressed in MF and correlated to detrimental clinical features in patients; therefore, we speculate that these lncRNAs could be involved in disease pathogenesis.

*GAS5* (Growth Arrest Specific 5) has been commonly considered as a tumor suppressor, being downregulated in tissues from several solid tumors (as reviewed by [47]). Nonetheless, in line with our results, circulating levels of *GAS5* were increased in plasma of patients affected by some tumors, like mesothelioma [48], and other pathological conditions, like osteoporosis [49].

The nuclear *MALAT1* (Metastasis-Associated Lung Adenocarcinoma Transcript 1) has been described to display contradictory action both as lncRNA promoting and suppressing metastasis [50,51]. 

**Figure 7:**
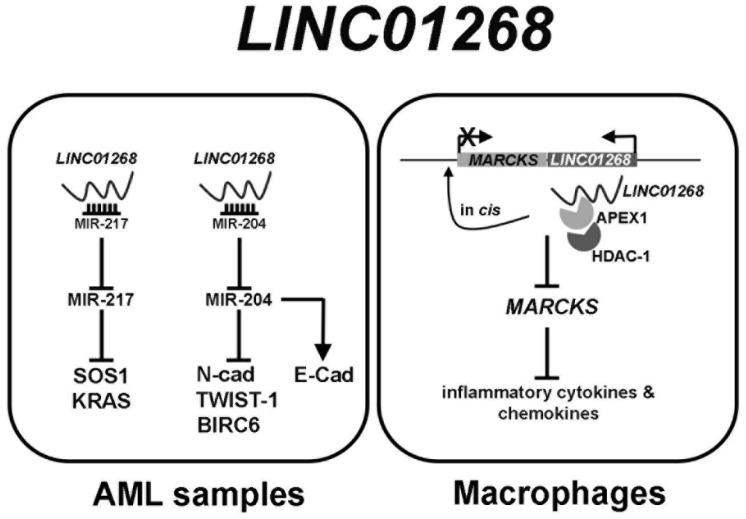
Graphical representation of *LINC01268* regulatory network.

*LINC01268* involvement in solid cancer is still poorly described and, to our knowledge, a description of circulating levels has not been assessed so far. Thus, we tried to design a network of gene expression regulation by *LINC01268*, which demonstrates that its overexpression is one of the mechanisms underlying leukemogenesis in AML and the induction of expression of inflammatory cytokines (Figure 7). 

*LINC01268* has been recently described as being upregulated in AML samples [52,53]. In a first work *LINC01268* has been described as acting as competing endogenous RNA (ceRNA) via sponging the onco-suppressor miR-217 which in turns inhibits at the post-transcriptional level *SOS1* mRNA. By modulating miR-217/SOS1 regulatory axis, *LINC01268* promotes cell growth and inhibits apoptosis [52]. The downregulation of miR-217 in AML cells was described also by Xiao et al., being proposed acting as a tumor suppressor by directly targeting *KRAS* [54].

An upregulation of *LINC01268* (therein referred as *LOC285758*) was described also to induce invasion and viability in AML cells by directly sponging miR-204, which inhibited *N-Cadherin* and *TWIST*-1 and increased E-Cadherin [53]. A downregulation of miR-204 in AML was also described by the work of Butrym et al., where decreased serum level of miR-204 have been correlated with a poor prognosis of AML [55]. Once again, miR-204 has been described to exert an anti-apoptotic effect by inhibiting *BIRC6* mRNA in AML cells [56]. Beyond its putative involvement in cancer pathogenesis, *LINC01268* (also known as *lnc-MARCKS* or *ROCKI*) acts as a master regulator of inflammatory response in macrophages, inducing pro-inflammatory cytokines and chemokines by acting in cis on *MARCKS* promoter [57].

## 5. Conclusions

As a whole, this work demonstrated that increased levels of circulating *LINC01268*, *GAS5* or *MALAT1* are associated with a number of clinical and molecular detrimental features and correlate with an inferior OS in MF patients. Notably, multivariate analysis confirmed that *LINC01268* plasma levels can be considered an independent variable. If the prognostic value of *LINC01268* is confirmed in future in an independent cohort, it could be evaluated in a perspective clinical study and then possibly integrated in contemporary prognostic models. 

To our knowledge, this is the first study describing the profile of circulating lncRNAs in plasma of MF patients and focusing on their putative role as biomarkers in clinical practice. In addition, our work sets the basis for further studies regarding the mechanism(s) underlying the role of these lncRNA(s) in MF pathogenesis.

## Figures and Tables

**Table 1:** Clinical and molecular features of MF patients included in our dataset, grouped according to the levels of single circulating lncRNAs. Data in the table are reported as n (%). *p* = *p*-value. N = evaluable samples. LDH = lactate dehydrogenase; DIPSS = Dynamic International Prognostic Score System. Significant *p*-value (*p* < 0.05) are represented in bold. “—” = missing value.

	*LINC01268*			*MALAT1*			*GAS5*			*TUG1*			*LINC00899*		
Variable	Low	High	*p*	Low	High	*p*	Low	High	*p*	Low	High	*p*	Low	High	*p*
**Males** (n evaluable, total = 134)	34 (56.67%)	41 (55.41%)	>0.9999	37 (53.62%)	38 (58.46%)	0.6048	38 (57.58%)	37 (54.41%)	0.7310	39 (59.09%)	36 (52.94%)	0.4916	39 (59.09%)	36 (52.94%)	0.4916
**Age**. Median, y (n evaluable, total = 135)	62.5	66.0	**0.0208**	65.0	65.5	0.2762	64.5	66.0	0.1084	65.0	65.0	0.537	65.0	65.0	0.5366
**Hemoglobin (Hb)** (n evaluable, total = 126)															
Median, g/L	11.75	11.00	0.1475	11.90	10.90	**0.0139**	11.95	10.75	**0.0072**	11.30	11.20	0.9622	11.20	11.20	0.5025
<10 g/L	12 (21.43%)	19 (27.14%)	0.5349	13 (20.31%)	18 (29.03%)	0.4058	12 (20.00%)	19 (28.79%)	0.3030	14 (22.58%)	17 (26.56%)	0.6811	12 (19.67%)	19 (29.23%)	0.2236
**Hematocrit (HCT)** (n evaluable, total = 99)	36.00	33.80	0.1051	38	34.95	**0.0386**	37.75	34.90	0.1026	34.95	35.9	0.9819	35.9	34.95	0.6925
**Leukocytes** (n evaluable, total = 120)															
Median, × 10^9^/L	7.60	11.00	**0.0012**	7.48	11.50	**<0.0001**	7.42	12.21	**<0.0001**	8.00	10.90	**0.0011**	8.05	9.29	**0.0390**
>25 × 10^9^/L	4 (7.14%)	13 (18.57%)	0.0621	7 (10.94%)	10 (16.13%)	0.3938	4 (6.67%)	13 (19.70%)	**0.0325**	4 (6.45%)	13 (20.31%)	**0.0228**	5 (8.20%)	12 (18.46%)	0.1123
**Platelets** (n evaluable, total = 120)															
Median, × 10^9^/L	439.00	246.00	**0.0021**	402.00	267.00	0.1364	492.00	246.00	**<0.0001**	406.00	267.00	0.0886	354.00	267.00	0.0835
<100 × 10^9^/L	7 (12.50%)	9 (12.86%)	>0.9999	10 (15.63%)	6 (9.68%)	0.4241	5 (8.33%)	11 (16.67%)	0.1887	8 (12.90%)	8 (12.50%)	>0.9999	6 (9.84%)	10 (15.38%)	0.4273
**Circulating** CD34 × 10^6^/L (n evaluable, total = 80)	2.00	35.00	**<0.0001**	1.30	63.30	**<0.0001**	1.20	68.30	**<0.0001**	2.00	29.00	**0.0002**	3.13	22.40	**0.0026**
**Constitutional symptoms** (n evaluable, total = 134)	18 (30.00%)	29 (39.19%)	0.2815	18 (26.09%)	29 (44.62%)	**0.0302**	17 (25.76%)	30 (44.12%)	**0.0306**	24 (36.36%)	23 (33.82%)	0.8567	22 (33.33%)	25 (36.76%)	0.7197
**Splenomegaly** (n evaluable, total = 128)	30 (51.72%)	56 (80.00%)	**0.0012**	31 (46.97%)	55 (88.71%)	**<0.0001**	33 (52.38%)	53 (81.54%)	**0.0006**	38 (61.29%)	48 (72.73%)	0.1907	39 (63.93%)	47 (70.15%)	0.5721
**LDH** (n evaluable, total = 104)	317	604	**0.0002**	338.00	716.00	**<0.0001**	362.00	604.00	**0.0004**	382	592	**0.0008**	382	701	**0.0001**
**Thrombosis** (n evaluable, total = 133)	13 (22.03%)	13 (17.57%)	0.6604	15 (22.06%)	11 (16.92%)	0.5159	13 (20.00%)	13 (19.12%)	>0.9999	15 (23.08%)	11 (16.18%)	0.3838	11 (16.92%)	15 (22.06%)	0.5159
**Bleeding** (n evaluable, total = 132)	6 (10.17%)	10 (13.70%)	0.6005	7 (10.45%)	9 (13.85%)	0.6020	7 (10.77%)	9 (13.43%)	0.7910	6 (9.38%)	10 (14.71%)	0.4284	5 (7.81%)	11 (16.18%)	0.1848
**Disease** (n evaluable, total = 119)															
Pre-PMF	24 (42.11%)	15 (20.83%)		32 (47.06%)	7 (11.48%)		28 (42.42%)	11 (17.46%)		24 (36.92%)	15 (23.44%)		21 (32.31%)	18 (28.13%)	
Overt PMF	16 (28.07%)	29 (40.28%)		15 (22.06%)	30 (49.18%)		19 (28.79%)	26 (41.27%)		21 (32.31%)	24 (37.50%)		25 (38.46%)	20 (31.25%)	
PET-MF	9 (15.79%)	15 (20.83%)		11 (16.18%)	13 (21.31%)		11 (16.67%)	13 (20.63%)		10 (15.38%)	14 (21.88%)		10 (15.38%)	14 (21.88%)	
PPV-MF	8 (14.04%)	13 (18.06%)	0.0761	10 (14.71%)	11 (18.03%)	**0.0001**	8 (12.12%)	13 (20.63%)	**0.0204**	10 (15.38%)	11 (17.19%)	0.3942	9 (13.85%)	12 (18.75%)	0.5990
**Fibrosis grade** ≥ 2 (n evaluable, total = 129)	33 (56.90%)	55 (77.46%)	**0.0144**	35 (52.24%)	53 (85.48%)	**<0.0001**	36 (55.38%)	52 (81.25%)	**0.0023**	39 (75.38%)	49 (60.94%)	0.0908	42 (65.63%)	46 (70.77%)	0.5740
**Driver mutation** (n evaluable, total = 133)															
*JAK2*	42 (70.00%)	39 (53.42%)	0.0737	46 (66.67%)	35 (54.69%)	0.2131	40 (61.54%)	41 (60.29%)	>0.9999	41 (63.08%)	40 (58.82%)	0.7225	41 (63.08%)	40 (58.82%)	0.7225
*MPL*	4 (6.67%)	4 (5.48%)	>0.9999	4 (5.80%)	4 (6.25%)	>0.9999	4 (6.15%)	4 (5.88%)	>0.9999	6 (9.23%)	2 (2.94%)	0.1587	4 (6.15%)	4 (5.88%)	>0.9999
*CALR*	10 (16.67%)	26 (35.62%)	**0.0184**	14 (20.29%)	22 (34.38%)	0.0804	17 (26.15%)	19 (27.94%)	0.8474	12 (18.46%)	24 (35.29%)	**0.0331**	17 (26.15%)	19 (27.94%)	0.8474
*TN*	4 (6.67%)	4 (5.48%)	>0.9999	5 (7.25%)	3 (4.69%)	0.7196	4 (6.15%)	4 (5.88%)	>0.9999	6 (9.23%)	2 (2.94%)	0.1587	3 (4.62%)	5 (7.35%)	0.7186
**High risk mutations** (n evaluable, total = 112)															
≥ 1	9 (19.15%)	28 (47.08%)	**0.0087**	13 (22.41%)	24 (44.44%)	**0.0163**	13 (24.07%)	24 (41.34%)	0.0703	16 (29.63%)	21 (36.21%)	0.5477	17 (30.91%)	20 (35.09%)	0.6907
≥ 2	0 (0.00%)	9 (13.85%)	—	2 (3.45%)	7 (12.96%)	0.0860	2 (3.70%)	7 (12.07%)	0.1643	3 (5.56%)	6 (10.34%)	0.4921	4 (7.27%)	5 (8.77%)	>0.9999
**High-risk mutations** (n evaluable, total = 105)															
*ASXL1*	6 (13.64%)	25 (40.98%)	**0.0025**	10 (18.18%)	21 (42.00%)	**0.0101**	9 (18.00%)	22 (40.00%)	**0.0183**	11 (21.57%)	20 (37.04%)	0.0917	14 (28.00%)	17 (30.91%)	0.8316
*EZH2*	1 (2.17%)	6 (9.52%)	0.2349	2 (3.45%)	5 (9.80%)	0.2486	2 (3.70%)	5 (9.09%)	0.4376	3 (5.56%)	4 (7.27%)	>0.9999	2 (3.70%)	5 (9.09%)	0.4376
*IDH1/2*	0 (0.00%)	4 (6.35%)	0.1364	1 (1.72%)	3 (5.88%)	0.3383	2 (3.64%)	2 (3.70%)	>0.9999	2 (3.70%)	2 (3.64%)	>0.9999	3 (5.56%)	1 (1.82%)	0.3634
*SRSF2*	2 (4.35%)	4 (6.35%)	>0.9999	3 (5.17%)	3 (5.88%)	>0.9999	3 (5.56%)	3 (5.45%)	>0.9999	4 (7.41%)	2 (3.64%)	0.4376	3 (5.56%)	3 (5.45%)	>0.9999
**DIPSS** (n evaluable, total = 130)															
Low	20 (33.90%)	5 (7.04%)		18 (27.27%)	7 (10.94%)		17 (26.56%)	8 (12.12%)		13 (20.63%)	12 (17.91%)		15 (23.44%)	10 (15.15%)	
Intermediate-1	18 (30.51%)	25 (35.21%)		27 (40.90%)	16 (25.00%)		29 (45.31%)	14 (21.21%)		23 (36.51%)	20 (29.85%)		21 (32.81%)	22 (33.33%)	
Intermediate-2	16 (27.12%)	25 (35.21%)		17 (25.76%)	24 (37.50%)		14 (21.88%)	27 (40.90%)		18 (28.57%)	23 (34.33%)		21 (31.81%)	20 (30.30%)	
High	5 (8.47%)	16 (22.54%)	**0.0007**	4 (6.06%)	17 (26.56%)	**0.0008**	4 (6.25%)	17 (25.76%)	**0.0001**	9 (14.29%)	12 (17.91%)	0.7613	7 (10.94%)	14 (21.21%)	0.3406
**Death** (n evaluable, total = 133)	14 (25.45%)	36 (51.43%)	**0.0035**	20 (32.26%)	32 (48.48%)	**0.0436**	17 (28.33%)	33 (50.77%)	**0.0115**	22 (44.00%)	28 (42.42%)	0.5876	27 (44.26%)	23 (35.94%)	0.3663
**AML transformation** (n evaluable, total = 133)	1 (1.92%)	10 (14.71%)	**0.0225**	5 (8.20%)	6 (10.17%)	0.7605	4 (6.90%)	7 (11.29%)	0.5317	3 (5.36%)	8 (12.50%)	0.2164	3 (5.00%)	8 (13.33%)	0.2043

**Table 2:** Results of multivariate regression analysis for overall survival and risk of transformation into acute myeloid leukemia. Analysis of the prognostic impact of high plasma level of each lncRNA was performed after stratification of samples according to DIPSS risk category. Significant *p*-value (*p* < 0.05) are represented in bold.

	Overall Survival		Transformation into Acute Leukemia	
	Hazard Ratio (95%CI)	*p*	Hazard Ratio (95%CI)	*p*
**High *LINC01268***	2.104 (1.08—4.12)	**0.0297**	8.190 (1.02—65.78)	**0.0479**
**High *MALAT1***	1.213 (0.66—2.23)	0.5343	1.792 (0.41—7.81)	0.4374
**High *GAS5***	1.742 (0.91—3.33)	0.09348	1.492 (0.40—5.56)	0.5507

## Data Availability

Data are contained within the article or Supplementary Material.

## Supplementary Materials

The following are available online at www.mdpi.com/article/10.3390/cancers13194744/s1, Table S1: List of lncRNAs whose expression has been evaluated in CD34+ cells, Table S2: List of lncRNAs evaluated in plasma samples, Table S3: Clinical and molecular features of MF patients included in our dataset, grouped according levels of *NEAT1* and *CDKN2B-AS1,* Figure S1: Analysis of stability of putative endogenous controls, Figure S2: Distributions of circulating lncRNAs in MF patients, Figure S3: Kaplan-Meier estimates of Overall Survival and Leukemia-free Survival of MF patients in the study.

## References

[1] Arber D.A., Orazi A., Hasserjian R., Thiele J., Borowitz M.J., Le Beau M.M., Bloomfield C.D., Cazzola M., Vardiman J.W. (2016). The 2016 Revision to the World Health Organization Classification of Myeloid Neoplasms and Acute Leukemia. Blood.

[2] Barosi G., Mesa R.A., Thiele J., Cervantes F., Campbell P.J., Verstovsek S., Dupriez B., Levine R.L., Passamonti F., On behalf of the International Working Group for Myelofibrosis Research and Treatment (IWG-MRT) (2008). Proposed Criteria for the Diagnosis of Post-Polycythemia Vera and Post-Essential Thrombocythemia Myelofibrosis: A Consensus Statement from the International Working Group for Myelofibrosis Research and Treatment. Leukemia.

[3] Tefferi A., Pardanani A. (2015). Myeloproliferative Neoplasms: A Contemporary Review. JAMA Oncol..

[4] Tefferi A., Vannucchi A.M. (2017). Genetic Risk Assessment in Myeloproliferative Neoplasms. Mayo Clin. Proc..

[5] Parenti S., Rontauroli S., Carretta C., Mallia S., Genovese E., Chiereghin C., Peano C., Tavernari L., Bianchi E., Fantini S. (2021). Mutated Clones Driving Leukemic Transformation Are Already Detectable at the Single-Cell Level in CD34-Positive Cells in the Chronic Phase of Primary Myelofibrosis. npj Precis. Onc..

[6] Hibbin J.A., Njoku O.S., Matutes E., Lewis S.M., Goldman J.M. (1984). Myeloid Progenitor Cells in the Circulation of Patients with Myelofibrosis and Other Myeloproliferative Disorders. Br. J. Haematol..

[7] Cervantes F., Dupriez B., Pereira A., Passamonti F., Reilly J.T., Morra E., Vannucchi A.M., Mesa R.A., Demory J.-L., Barosi G. (2009). New Prognostic Scoring System for Primary Myelofibrosis Based on a Study of the International Working Group for Myelofibrosis Research and Treatment. Blood.

[8] Passamonti F., Cervantes F., Vannucchi A.M., Morra E., Rumi E., Pereira A., Guglielmelli P., Pungolino E., Caramella M., Maffioli M. (2010). A Dynamic Prognostic Model to Predict Survival in Primary Myelofibrosis: A Study by the IWG-MRT (International Working Group for Myeloproliferative Neoplasms Research and Treatment). Blood.

[9] Guglielmelli P., Lasho T.L., Rotunno G., Mudireddy M., Mannarelli C., Nicolosi M., Pacilli A., Pardanani A., Rumi E., Rosti V. (2018). MIPSS70: Mutation-Enhanced International Prognostic Score System for Transplantation-Age Patients With Primary Myelofibrosis. JCO.

[10] Tefferi A., Guglielmelli P., Lasho T.L., Gangat N., Ketterling R.P., Pardanani A., Vannucchi A.M. (2018). MIPSS70+ Version 2.0: Mutation and Karyotype-Enhanced International Prognostic Scoring System for Primary Myelofibrosis. JCO.

[11] Passamonti F., Giorgino T., Mora B., Guglielmelli P., Rumi E., Maffioli M., Rambaldi A., Caramella M., Komrokji R., Gotlib J. (2017). A Clinical-Molecular Prognostic Model to Predict Survival in Patients with Post Polycythemia Vera and Post Essential Thrombocythemia Myelofibrosis. Leukemia.

[12] Tefferi A. (2021). Primary Myelofibrosis: 2021 Update on Diagnosis, Risk-stratification and Management. Am. J. Hematol..

[13] Rontauroli S., Castellano S., Guglielmelli P., Zini R., Bianchi E., Genovese E., Carretta C., Parenti S., Fantini S., Mallia S. (2021). Gene Expression Profile Correlates with Molecular and Clinical Features in Patients with Myelofibrosis. Blood Adv..

[14] Kopp F., Mendell J.T. (2018). Functional Classification and Experimental Dissection of Long Noncoding RNAs. Cell.

[15] Cabili M.N., Trapnell C., Goff L., Koziol M., Tazon-Vega B., Regev A., Rinn J.L. (2011). Integrative Annotation of Human Large Intergenic Noncoding RNAs Reveals Global Properties and Specific Subclasses. Genes Dev..

[16] Statello L., Guo C.-J., Chen L.-L., Huarte M. (2021). Gene Regulation by Long Non-Coding RNAs and Its Biological Functions. Nat. Rev. Mol. Cell Biol..

[17] Wagner L.A., Christensen C.J., Dunn D.M., Spangrude G.J., Georgelas A., Kelley L., Esplin M.S., Weiss R.B., Gleich G.J. (2007). EGO, a Novel, Noncoding RNA Gene, Regulates Eosinophil Granule Protein Transcript Expression. Blood.

[18] Zhang X., Weissman S.M., Newburger P.E. (2014). Long Intergenic Non-Coding RNA HOTAIRM1 Regulates Cell Cycle Progression during Myeloid Maturation in NB4 Human Promyelocytic Leukemia Cells. RNA Biol..

[19] Alvarez-Dominguez J.R., Hu W., Gromatzky A.A., Lodish H.F. (2014). Long Noncoding RNAs during Normal and Malignant Hematopoiesis. Int. J. Hematol..

[20] Pennucci V., Zini R., Norfo R., Guglielmelli P., Bianchi E., Salati S., Sacchi G., Prudente Z., Tenedini E., Ruberti S. (2015). Abnormal Expression Patterns of *WT1-as, MEG3* and *ANRIL* Long Non-Coding RNAs in CD34+ Cells from Patients with Primary Myelofibrosis and Their Clinical Correlations. Leuk. Lymphoma.

[21] Anfossi S., Babayan A., Pantel K., Calin G.A. (2018). Clinical Utility of Circulating Non-Coding RNAs—an Update. Nat. Rev. Clin. Oncol..

[22] Gruner H.N., McManus M.T. (2021). Examining the Evidence for Extracellular RNA Function in Mammals. Nat. Rev. Genet..

[23] Pös O., Biró O., Szemes T., Nagy B. (2018). Circulating Cell-Free Nucleic Acids: Characteristics and Applications. Eur. J. Hum. Genet..

[24] Shah J.S., Soon P.S., Marsh D.J. (2016). Comparison of Methodologies to Detect Low Levels of Hemolysis in Serum for Accurate Assessment of Serum MicroRNAs. PLoS ONE.

[25] Livak K.J., Schmittgen T.D. (2001). Analysis of Relative Gene Expression Data Using Real-Time Quantitative PCR and the 2−ΔΔCT Method. Methods.

[26] Vandesompele J., Preter K.D., Roy N.V., Paepe A.D. (2002). Accurate Normalization of Real-Time Quantitative RT-PCR Data by Geometric Averaging of Multiple Internal Control Genes. Genome Biol..

[27] Alvarez-Dominguez J.R., Lodish H.F. (2017). Emerging Mechanisms of Long Noncoding RNA Function during Normal and Malignant Hematopoiesis. Blood.

[28] Li W., Ren Y., Si Y., Wang F., Yu J. (2018). Long Non-Coding RNAs in Hematopoietic Regulation. Cell Regen..

[29] Wang Y., Li Y., Song H.-Q., Sun G.-W. (2018). Long Non-Coding RNA LINC00899 as a Novel Serum Biomarker for Diagnosis and Prognosis Prediction of Acute Myeloid Leukemia. Eur. Rev. Med. Pharmacol. Sci..

[30] Cho S.-F., Chang Y.C., Chang C.-S., Lin S.-F., Liu Y.-C., Hsiao H.-H., Chang J.-G., Liu T.-C. (2014). MALAT1 Long Non-Coding RNA Is Overexpressed in Multiple Myeloma and May Serve as a Marker to Predict Disease Progression. BMC Cancer.

[31] Nobili L., Lionetti M., Neri A. (2016). Long Non-Coding RNAs in Normal and Malignant Hematopoiesis. Oncotarget.

[32] Kotzin J.J., Spencer S.P., McCright S.J., Kumar D.B.U., Collet M.A., Mowel W.K., Elliott E.N., Uyar A., Makiya M.A., Dunagin M.C. (2016). The Long Non-Coding RNA Morrbid Regulates Bim and Short-Lived Myeloid Cell Lifespan. Nature.

[33] Fallik N., Bar-Lavan Y., Greenshpan Y., Goldstein O., Grosch M., Drukker M., Gazit R. (2017). *Neat1* in Hematopoietic Stem Cells. Oncotarget.

[34] Cuadros M., Andrades Á., Coira I.F., Baliñas C., Rodríguez M.I., Álvarez-Pérez J.C., Peinado P., Arenas A.M., García D.J., Jiménez P. (2019). Expression of the Long Non-Coding RNA TCL6 Is Associated with Clinical Outcome in Pediatric B-Cell Acute Lymphoblastic Leukemia. Blood Cancer J..

[35] Vannucchi A.M., Lasho T.L., Guglielmelli P., Biamonte F., Pardanani A., Pereira A., Finke C., Score J., Gangat N., Mannarelli C. (2013). Mutations and Prognosis in Primary Myelofibrosis. Leukemia.

[36] Bao T., Davidson N.E. (2008). Gene Expression Profiling of Breast Cancer. Adv. Surg..

[37] Oliveira D.V.N.P., Prahm K.P., Christensen I.J., Hansen A., Høgdall C.K., Høgdall E.V. (2021). Gene Expression Profile Association with Poor Prognosis in Epithelial Ovarian Cancer Patients. Sci. Rep..

[38] Grossman D., Kim C.C., Hartman R.I., Berry E., Nelson K.C., Okwundu N., Curiel-Lewandrowski C., Leachman S.A., Swetter S.M. (2019). Prognostic Gene Expression Profiling in Melanoma: Necessary Steps to Incorporate into Clinical Practice. Melanoma Manag..

[39] Ng S.W.K., Mitchell A., Kennedy J.A., Chen W.C., McLeod J., Ibrahimova N., Arruda A., Popescu A., Gupta V., Schimmer A.D. (2016). A 17-Gene Stemness Score for Rapid Determination of Risk in Acute Leukaemia. Nature.

[40] Le Bousse-Kerdilès M.-C. (2012). Primary Myelofibrosis and the “Bad Seeds in Bad Soil” Concept. Fibrogenes. Tissue Repair.

[41] Orvain C., Luque Paz D., Dobo I., Cottin L., Le Calvez G., Chauveau A., Mercier M., Farhi J., Boyer F., Ianotto J.C. (2016). Circulating Cd34+ Cell Count Differentiates Primary Myelofibrosis from Other Philadelphia-Negative Myeloproliferative Neoplasms: A Pragmatic Study. Ann. Hematol..

[42] Lo K.-W., Lo Y.D., Leung S.-F., Tsang Y.-S., Chan L.Y., Johnson P.J., Hjelm N.M., Lee J.C., Huang D.P. (1999). Analysis of Cell-Free Epstein-Barr Virus-Associated RNA in the Plasma of Patients with Nasopharyngeal Carcinoma. Clin. Chem..

[43] Kopreski M.S., Benko F.A., Kwak L.W., Gocke C.D. (1999). Detection of Tumor Messenger RNA in the Serum of Patients with Malignant Melanoma. Clin. Cancer Res..

[44] Reis P.P., Drigo S.A., Carvalho R.F., Lopez Lapa R.M., Felix T.F., Patel D., Cheng D., Pintilie M., Liu G., Tsao M.-S. (2020). Circulating MiR-16-5p, MiR-92a-3p, and MiR-451a in Plasma from Lung Cancer Patients: Potential Application in Early Detection and a Regulatory Role in Tumorigenesis Pathways. Cancers.

[45] Penna D., Nicolosi M., Mudireddy M., Szuber N., Vallapureddy R., Lasho T.L., Finke C., Hanson C.A., Ketterling R.P., Pardanani A. (2018). 20+ Years and Alive with Primary Myelofibrosis: Phenotypic Signature of Very Long-Lived Patients. Blood.

[46] Tefferi A. (2016). Primary Myelofibrosis: 2017 Update on Diagnosis, Risk-stratification, and Management. Am. J. Hematol..

[47] Ji J., Dai X., Yeung S.-C.J., He X. (2019). The Role of Long Non-Coding RNA GAS5 in Cancers. CMAR.

[48] Weber D.G., Casjens S., Brik A., Raiko I., Lehnert M., Taeger D., Gleichenhagen J., Kollmeier J., Bauer T.T., The MoMar Study Group (2020). Circulating Long Non-Coding RNA GAS5 (Growth Arrest-Specific Transcript 5) as a Complement Marker for the Detection of Malignant Mesothelioma Using Liquid Biopsies. Biomark. Res..

[49] Visconti V.V., Fittipaldi S., Ciuffi S., Marini F., Isaia G., D’Amelio P., Migliaccio S., Marcocci C., Minisola S., Nuti R. (2020). Circulating Long Non-Coding RNA GAS5 Is Overexpressed in Serum from Osteoporotic Patients and Is Associated with Increased Risk of Bone Fragility. Int. J. Mol. Sci..

[50] Ji Q., Zhang L., Liu X., Zhou L., Wang W., Han Z., Sui H., Tang Y., Wang Y., Liu N. (2014). Long Non-Coding RNA MALAT1 Promotes Tumour Growth and Metastasis in Colorectal Cancer through Binding to SFPQ and Releasing Oncogene PTBP2 from SFPQ/PTBP2 Complex. Br. J. Cancer.

[51] Kim J., Piao H.-L., Kim B.-J., Yao F., Han Z., Wang Y., Xiao Z., Siverly A.N., Lawhon S.E., Ton B.N. (2018). Long Noncoding RNA MALAT1 Suppresses Breast Cancer Metastasis. Nat. Genet..

[52] Chen B., Li Y., Nie Y., Tang A., Zhou Q. (2020). Long Non-Coding RNA LINC01268 Promotes Cell Growth and Inhibits Cell Apoptosis by Modulating MiR-217/SOS1 Axis in Acute Myeloid Leukemia. Braz. J. Med. Biol. Res..

[53] Xue F., Che H. (2020). The Long Non-coding RNA LOC285758 Promotes Invasion of Acute Myeloid Leukemia Cells by Down-regulating MiR-204-5p. FEBS Open Bio.

[54] Xiao Y., Deng T., Su C., Shang Z. (2017). MicroRNA 217 Inhibits Cell Proliferation and Enhances Chemosensitivity to Doxorubicin in Acute Myeloid Leukemia by Targeting KRAS. Oncol. Lett..

[55] Butrym A., Rybka J., Baczyńska D., Tukiendorf A., Kuliczkowski K., Mazur G. (2015). Low Expression of MicroRNA-204 (MiR-204) Is Associated with Poor Clinical Outcome of Acute Myeloid Leukemia (AML) Patients. J. Exp. Clin. Cancer Res..

[56] Wang Z., Luo H., Fang Z., Fan Y., Liu X., Zhang Y., Rui S., Chen Y., Hong L., Gao J. (2018). MiR-204 Acts as a Potential Therapeutic Target in Acute Myeloid Leukemia by Increasing BIRC6-Mediated Apoptosis. BMB Rep..

[57] Zhang Q., Chao T., Patil V.S., Qin Y., Tiwari S.K., Chiou J., Dobin A., Tsai C., Li Z., Dang J. (2019). The Long Noncoding RNA*ROCKI* Regulates Inflammatory Gene Expression. EMBO J..

