# Supplementary material for: Increased Plasma Levels of lncRNAs LINC01268, GAS5 and MALAT1 Correlate with Negative Prognostic Factors in Myelofibrosis
Source: Cancers (Basel). 2021 Sep 22;13(19):4744. doi: 10.3390/cancers13194744 (PMC8507546; doi:10.3390/cancers13194744)
Supplement: Supplementary file 1 [file cancers-13-04744-s001.zip › cancers-1352336-supplementary.pdf]

Table S1. List of lncRNAs whose expression has been evaluated in CD34+ cells. For each lncRNA, we reported both the gene ID and the full gene name. Assay ID of Taqman probe exploited in OpenArray experiments was also listed.

Gene ID	Full gene name	Assay ID
SH3BP5-AS1	SH3BP5 antisense RNA 1	Custom Assay
HCP5	HLA complex P5	Hs00198533_g1
TCL6	T Cell Leukemia/Lymphoma 6	Hs00220956_m1
MEG3	Maternally Expressed Gene 3	Hs00292028_m1
LINC01013	Long intergenic non-protein coding RNA 1013	Hs00395149_m1
LINC01353	Long intergenic non-protein coding RNA 1353	Hs00418657_g1
IMPORTIN 8	Importin 8	Hs00914057_m1
TMEM9B-AS1	TMEM9B antisense RNA 1	Hs00937602_m1
LINC01578	Long intergenic non-protein coding RNA 1578	Hs00965389_g1
LINC01128	Long intergenic non-protein coding RNA 1128	Hs01005975_g1
HOXB-AS3	HOXB cluster antisense RNA 3	Hs01080705_g1
LINC01122	Long intergenic non-protein coding RNA 1122	Hs01376003_m1
EIF3J-AS1	EIF3J divergent transcript	Hs01387474_g1
LEF1-AS1	LEF1 antisense RNA 1	Hs01398290_m1
UBA6-AS1	UBA6 divergent transcript	Hs01591547_m1
LINC01572	Long intergenic non-protein coding RNA 1572	Hs01594211_mH
FAM170B-AS1	FAM170B antisense RNA 1	Hs01598107_m1
RPL19	Ribosomal Protein L19	Hs02338565_gH
GAPDH	Glyceraldehyde-3-phosphate dehydrogenase	Hs02786624_g1
HPRT1	Hypoxanthine Phosphoribosyltransferase 1	Hs02800695_m1
DGCR11	DiGeorge syndrome critical region gene 11	Hs03654138_s1
LINC00114	Long intergenic non-protein coding RNA 114	Hs03664994_m1
N4BP2L2-IT2	N4BPL2 intronic transcript 2	Hs03666925_s1
DHRS4-AS1	DHRS4 antisense RNA 1	Hs03674802_s1
OIP5-AS1	OIP5 antisense RNA 1	Hs03677189_g1
LINC00899	Long intergenic non-protein coding RNA 899	Hs03678297_mH
LINC00426	Long intergenic non-protein coding RNA 426	Hs03680869_m1
PSMD6-AS2	PSMD6 antisense RNA 2	Hs03838639_s1
PSMB8-AS1	PSMB8 antisense RNA 1 (head to head)	Hs04232145_g1
PSMB8_AS1	PSMB8 antisense RNA 1 (head to head)	Hs04232146_m1
PSMB8_AS1	PSMB8 antisense RNA 1 (head to head)	Hs04232148_m1
BACE1 AS	BACE1 antisense RNA	Hs04232267_s1
OLMALINC	oligodendrocyte maturation-associated long intergenic non-coding RNA	Hs04233809_s1
SLC8A1-AS1	SLC8A1 antisense RNA 1	Hs04274406_m1
LINC01268	Long intergenic non-protein coding RNA 1268	Hs04274455_g1
LINC01128	Long intergenic non-protein coding RNA 1128	Hs04332526_m1
NEBL-AS1	NEBL antisense RNA 1	Hs04333166_s1
LINC01507	Long intergenic non-protein coding RNA 1507	Hs04406631_m1
LINC01410	Long intergenic non-protein coding RNA 1410	Hs04406762_m1
HOXB-AS3	HOXB cluster antisense RNA 3	Hs04408889_s1
LYRM4-AS1	LYRM4 antisense RNA 1	Hs04409446_s1
CARD8-AS1	CARD8 antisense RNA 1	Hs04409473_g1
LINC01296	Long intergenic non-protein coding RNA 1296	Hs04942686_m1
LOC389641	TNFRSF10A divergent transcript	Hs05047639_s1
LOXL1-AS1	LOXL1 antisense RNA 1	Hs05051255_s1
PRKCQ-AS1	PRKCQ antisense RNA 1	Hs05054777_s1
GAPDH	Glyceraldehyde-3-phosphate dehydrogenase	Hs99999905_m1

**LINC00408**	Long intergenic non-protein coding RNA 408	Hs01388535_g1
**LYRM4-AS1**	LYRM4 antisense RNA 1	Hs04407449_m1
**LINC01296**	Long intergenic non-protein coding RNA 1296	Custom Assay
**LINC00891**	Long intergenic non-protein coding RNA 891	Custom Assay
**DGCR2**	DiGeorge syndrome critical region gene 2	Custom Assay
**LINC01374**	Long intergenic non-protein coding RNA 1374	Custom Assay

Table S2. List of lncRNAs evaluated in plasma samples. For each lncRNA, we reported both the gene ID and the full gene name. The different isoforms detected by single assays were indicated with NR_ accession prefix, according to the curated Refseq records. Median fold change obtained by qRT-PCR experiments is indicated in the last column.

Gene ID	Full Gene name	Assay ID	Detected ISoforms	Fold change
LINC00899	Long intergenic non-protein coding RNA 899	Hs03678297_mH	NR_027036	9.5731
LINC01268 (MROCKI)	Long intergenic non-protein coding RNA 1268 (Master Regulator of Cytokines and Inflammation)	Hs04274455_g1	NR_038863	2164.773
GAS5	Growth Arrest Specific transcript 5	Hs03464472_m1	NR_002578	1.8200
MALAT1	Metastasis Associated Lung Adenocarcinoma Transcript 1	Hs00273907_s1	NR_002819	5.2343
TUG1	Taurine upregulated gene 1	Hs00215501_m1	NR_002323; NR_110493	7.8953
NEAT1	Nuclear Paraspeckle Assembly Transcript 1	Hs03453535_s1	NR_028272; NR_131012	4.0418
CDKN2B-AS1 (ANRIL)	CDKN2B Antisense RNA 1 (Antisense Non-coding RNA in the INK4 Locus)	Hs03300540_m1	NR_047533; NR_003529; NR_047532; NR_047534; NR_047535; NR_047536; NR_047537; NR_047538; NR_047539; NR_047540; NR_047541; NR_047542; NR_047543; NR_120536 ;	22011.96
MIR4435-2HG	MIR4435-2 Host Gene (myeloid RNA regulator of Bim-induced death)	Hs03680374_m1	NR_015395; NR_136161; NR_136162; NR_136163; NR_136164; NR_136166	1.2303
MIR155-HG (BIC)	MIR155 Host Gene (B-cell Integration Cluster)	Hs01374569_m1	NR_001458	1.6976
HOXB-AS3	HOXB cluster antisense RNA 3	Hs00420340_m1	NR_033202; NR_033203; NR_033205	1.5562
HOTAIRM1	HOXA Transcript Antisense RNA, Myeloid-Specific 1	Hs03296533_g1	NR_038366	1.1057
MEG3	Maternally Expressed Gene 3	Hs00292028_m1	NR_002766; NR_003530; NR_003531; NR_033358; NR_033359; NR_033360; NR_046464; NR_046465; NR_046466; NR_046467; NR_046469; NR_046470; NR_046471; NR_046472; NR_046473	-
TCL6	T Cell Leukemia/Lymphoma 6	Hs00220956_m1	NR_028288	-
H19	H19 Imprinted Maternally Expressed Transcript	Hs00399294_g1	NR_002196; NR_131223; NR_131224	-

Table S3. Clinical and molecular features of MF patients included in our dataset, grouped according to levels of *NEAT1* and *CDKN2B-AS1*. Data in the table are reported as n (%). P = p-value. N = evaluable samples. LDH = lactate dehydrogenase. DIPSS = Dynamic Internation Prognostic Score System. Significant p-value ($P < .05$) are represented in bold. "—" = missing value.

	NEAT1			*CDKN2B-AS1*		
**Variable**	**Low**	**High**	***P***	**Low**	**High**	***P***
**Males** (n evaluable, total = 134)	42 (61.76%)	33 (50.00%)	0.2231	33 (64.71%)	42 (50.60%)	0.1514
**Age**, median, y, (n evaluable, total = 135)	64.0	67.0	0.1656	65.5	65.0	0.918
**Hemoglobin (Hb)** (n evaluable, total = 126)						
Median, g/L	11.50	11.10	0.9351	11.30	11.20	0.8703
<10 g/L	15 (23.81%)	16 (25.40%)	>0.9999	13 (26.53%)	18 (23.38%)	0.8323
**Hematocrit (HCT)** (n evaluable, total = 99)	35.1	36	0.9431	35.3	35.25	0.9622
**Leukocytes** (n evaluable, total = 120)						
Median, x 10^9/L	8.32	8.75	0.2821	7.48	8.90	0.0865
>25 x 10^9/L	9 (14.29%)	8 (12.70%)	0.7943	6 (12.24%)	11 (14.29%)	0.7438
**Platelets** (n evaluable, total = 120)						
Median, x 10^9/L	284.00	308.00	0.1010	289.00	301.00	0.2224
<100 x 10^9/L	11 (17.46%)	5 (7.94%)	0.1797	7 (14.29%)	9 (11.69%)	0.7851
**Circulating CD34** x 10^6/L (n evaluable, total = 80)	5.00	11.00	0.3378	9.80	6.20	0.9765
**Constitutional symptoms** (n evaluable, total = 134)	19 (27.94%)	28 (42.42%)	0.1032	18 (35.29%)	29 (34.94%)	>0.9999
**Splenomegaly** (n evaluable, total = 128)	42 (64.62%)	44 (69.84%)	0.5754	33 (66.00%)	53 (67.95%)	0.8487
**LDH** (n evaluable, total = 104)	362	605	**0.0005**	496.5	466	0.7698
**Thrombosis** (n evaluable, total = 133)	16 (23.88%)	10 (15.15%)	0.2746	13 (25.49%)	13 (15.85%)	0.1849
**Bleeding** (n evaluable, total = 132)	9 (13.43%)	7 (10.77%)	0.7910	5 (10.00%)	11 (13.41%)	0.7842
**Disease** (n evaluable, total = 119)						
Pre-PMF	22 (33.85%)	17 (26.56%)		15 (30.61%)	24 (30.00%)	
Overt PMF	20 (30.77%)	25 (39.06%)		19 (38.76%)	26 (32.50%)	
PET-MF	11 (16.92%)	13 (20.31%)		10 (20.41%)	14 (17.50%)	
PPV-MF	12 (18.46%)	9 (14.06%)	0.6184	5 (10.20%)	16 (20.00%)	0.5171
**Fibrosis grade≥2** (n evaluable, total = 129)	42 (64.62%)	46 (71.88%)	0.4505	34 (69.39%)	54 (67.50%)	0.8482
**Driver mutation** (n evaluable, total = 133)						
JAK2	45 (66.18%)	36 (55.38%)	0.2182	34 (57.32%)	47 (66.67%)	0.3612
MPL	7 (10.29%)	1 (1.54%)	0.0627	2 (3.92%)	6 (7.32%)	0.7100
CALR	11 (16.18%)	25 (38.46%)	**0.0059**	13 (25.49%)	23 (28.05%)	0.8419
TN	5 (7.35%)	3 (4.62%)	0.7186	2 (3.92%)	6 (7.32%)	0.7100
**High risk mutations** (n evaluable, total = 112)						
≥ 1	21 (37.50%)	16 (28.57%)	0.4219	15 (36.59%)	22 (30.99%)	0.6770
≥ 2	6 (10.71%)	3 (5.36%)	0.4893	5 (12.20%)	4 (5.63%)	0.2837
**High-risk mutations** (n evaluable, total = 105)						
ASXL1	16 (31.37%)	15 (27.78%)	0.8308	12 (29.27%)	19 (29.69%)	>0.9999
EZH2	4 (7.27%)	3 (5.56%)	>0.9999	4 (9.76%)	3 (4.41%)	0.4218
IDH1/2	3 (5.45%)	1 (1.85%)	0.6180	3 (7.32%)	1 (1.47%)	0.1485
SRSF2	6 (10.91%)	0 (0.00%)	**0.0271**	2 (4.88%)	4 (5.88%)	>0.9999
**DIPSS** (n evaluable, total = 130)						
Low	11 (16.92%)	14 (21.54%)		9 (18.00%)	16 (20.00%)	
Intermediate-1	25 (38.46%)	18 (27.69%)		19 (38.00%)	24 (30.00%)	
Intermediate-2	21 (32.31%)	20 (30.77%)		14 (28.00%)	27 (33.75%)	
High	8 (12.31%)	13 (20.00%)	0.4378	8 (16.00%)	13 (16.25%)	0.8053
**Death** (n evaluable, total = 133)	32 (52.42%)	22 (34.38%)	**0.0483**	24 (48.98%)	30 (39.47%)	0.356
**AML transformation** (n evaluable, total = 133)	5 (7.46%)	6 (9.09%)	0.7636	4 (7.84%)	7 (8.54%)	>0.9999

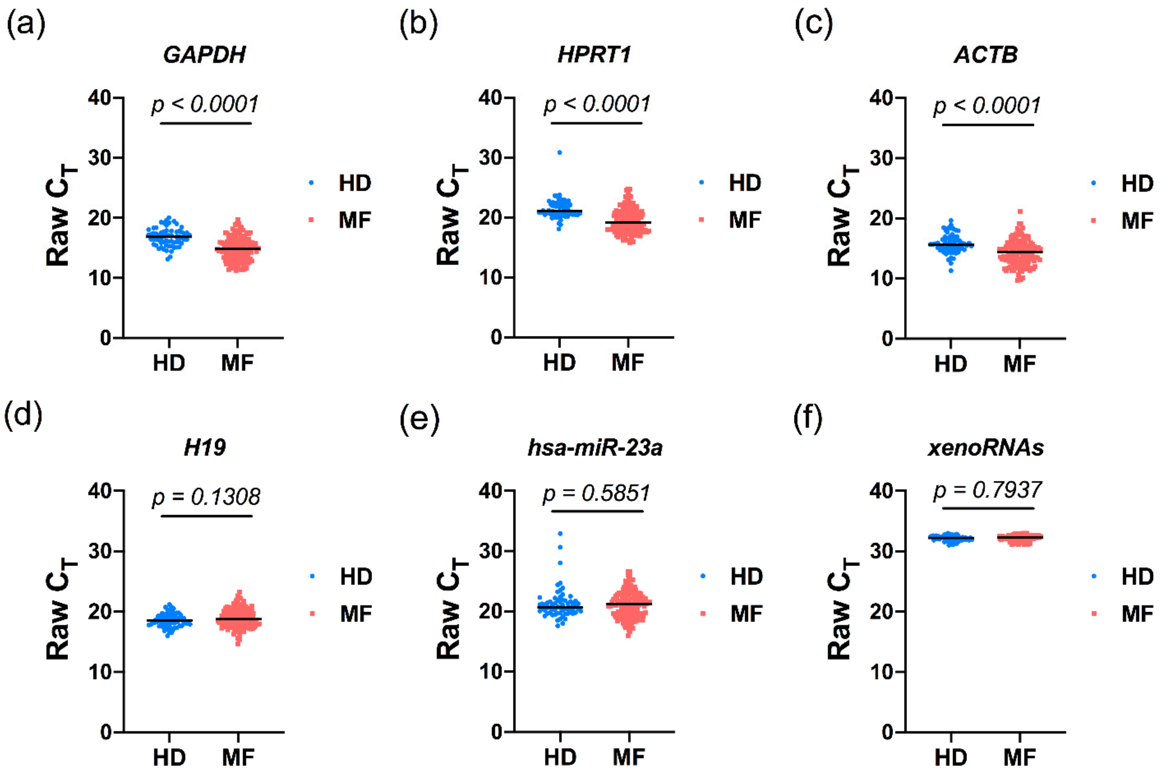

Figure S1. – Analysis of stability of putative endogenous controls. Scatter dot plots represent the raw C_T values in HD and MF samples of RNAs selected as putative endogenous control. xenoRNAs have been evaluated as exogenous control. HD samples are represented by blue dots whereas MF sample by red dots. Black horizontal line represents the median value. P = *p*-value computed by non-parametric Mann-Whitney U test. N = 65 for control samples; n = 143 for MF samples. HD = healthy donor, MF = myelofibrosis samples.

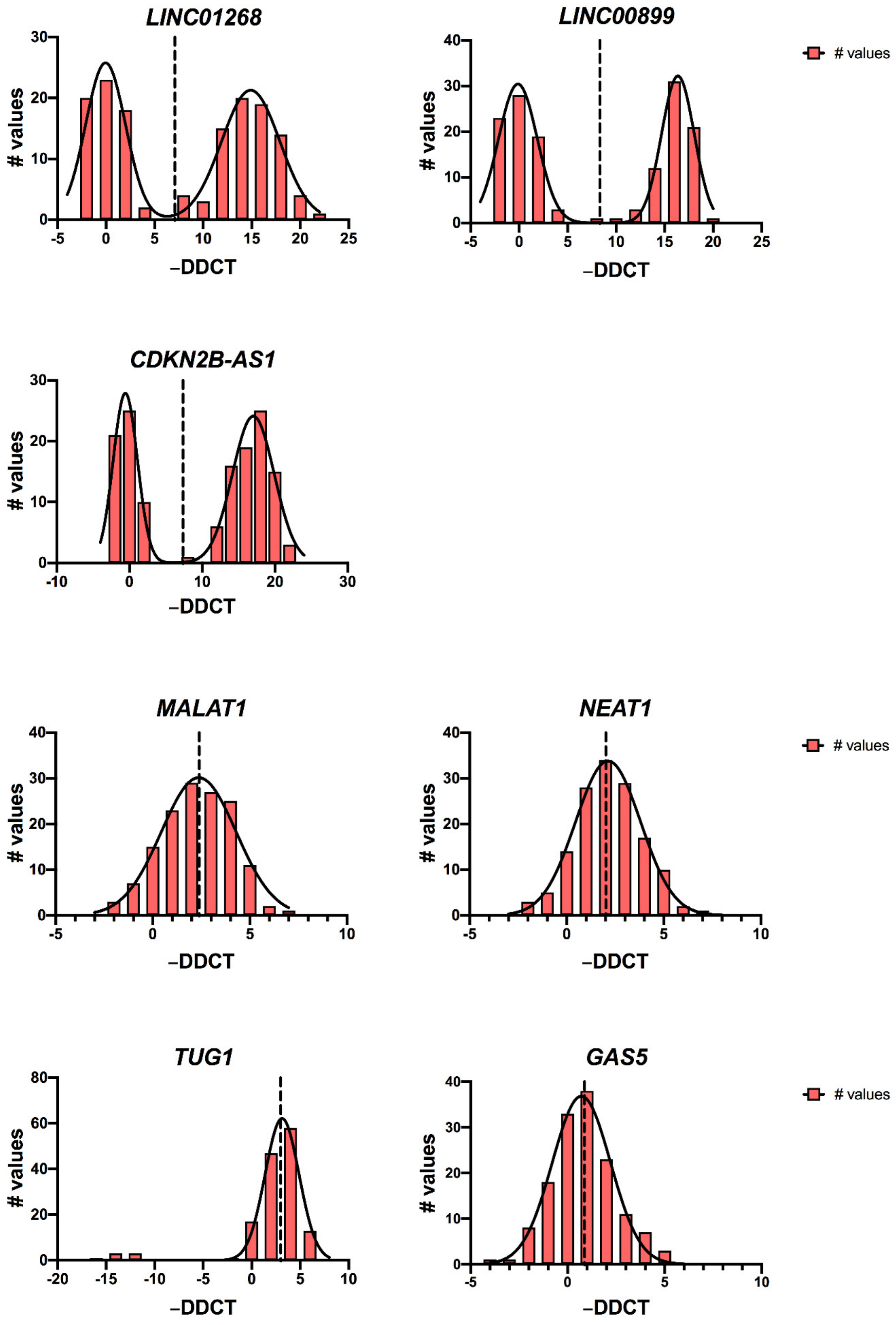

Figure S2. Distributions of circulating lncRNAs in MF patients. Histograms displaying frequency distribution of the circulating levels of the different lncRNAs in MF patients. The best-fitting curve was superimposed to the histograms to indicate the type of distribution on the samples. A vertical dashed line represents the cut-point exploited to split cohort of patients in two separate groups (low or high).

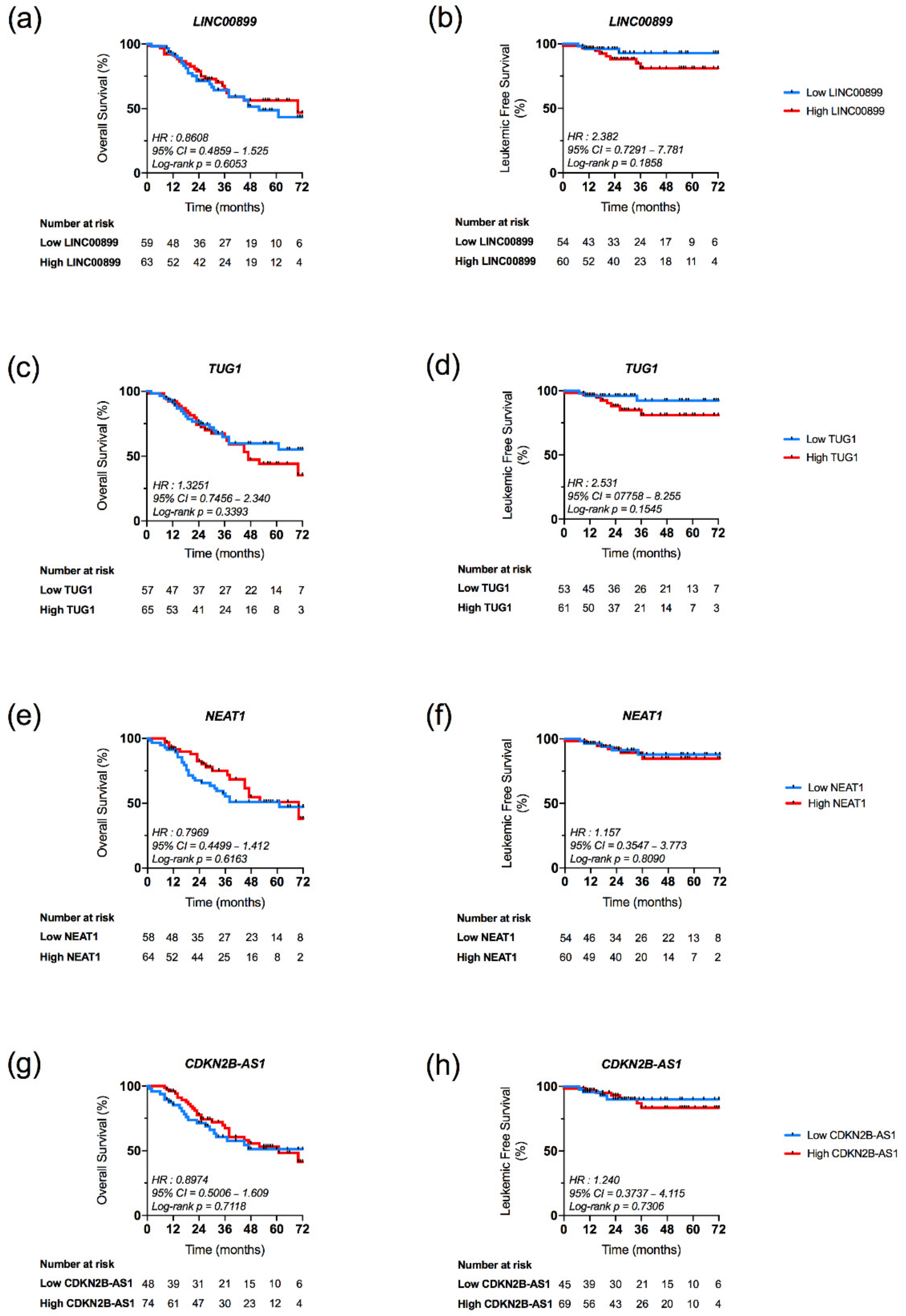

Figure 3. Kaplan-Meier estimates of Overall Survival and Leukemia-free Survival of MF patients in the study. Patients cohort was stratified into two groups (low and high) according to the plasma levels of target lncRNA, as described in the text. Differences between two survival curve was evaluated by Log-rank (Mantel-Cox) test. Blue and red curves represent patients with low of high levels of circulating target, respectively. HR = hazard ratio computed to determine the magnitude of differences between two curves. *P*-value was computed by log-rank test. 95% CI = 95% confidence interval.
